# Amino acid competition shapes *Acinetobacter baumannii* gut carriage

**DOI:** 10.1016/j.chom.2025.07.003

**Published:** 2025-08-04

**Authors:** Xiaomei Ren, R. Mason Clark, Dziedzom A. Bansah, Elizabeth N. Varner, Connor R. Tiffany, Kanchan Jaswal, John H. Geary, Olivia A. Todd, Jonathan D. Winkelman, Elliot S. Friedman, Riley N. Jarrett, Babette S. Zemel, Gary D. Wu, Joseph P. Zackular, William H. DePas, Judith Behnsen, Lauren D. Palmer

**Affiliations:** 1 Department of Microbiology and Immunology, University of Illinois Chicago, Chicago, IL 60612, USA; 2 Department of Pediatrics, University of Pittsburgh School of Medicine, Pittsburgh, PA 15227, USA; 3 Division of Protective Immunity, Children’s Hospital of Philadelphia, Philadelphia, PA 19104, USA; 4 Trestle LLC, Milwaukee, WI 53211, USA; 5 Division of Gastroenterology and Hepatology, Perelman School of Medicine, University of Pennsylvania, Philadelphia, PA 19104, USA; 6 Department of Pediatrics, Perelman School of Medicine, University of Pennsylvania, Philadelphia, PA 19104, USA; 7 Division of Gastroenterology, Hepatology, and Nutrition, Children’s Hospital of Philadelphia, Philadelphia, PA 19104, USA; 8 Department of Pathology and Laboratory Medicine, Perelman School of Medicine, University of Pennsylvania, Philadelphia, PA 19104, USA; 9 Center for Microbial Medicine, Children’s Hospital of Philadelphia, Philadelphia, PA 19104, USA; 10 Present address: American University of the Caribbean, Cupecoy, Sint Maarten; 11 These authors contributed equally; 12 Lead contact

## Abstract

Asymptomatic colonization is often critical for persistence of antimicrobial-resistant pathogens, such as *Acinetobacter baumannii*, and can increase the risk of clinical infections. However, the ecological factors shaping *A. baumannii* gut colonization remain unclear. We show that *A. baumannii* and other pathogenic *Acinetobacter* evolved to utilize the amino acid ornithine, a non-preferred carbon source, to compete with resident microbiota and persist in the gut in mice. *A. baumannii* encodes ornithine succinyltransferase (AstO) necessary for catabolizing ornithine, especially in conditions of increased microbial diversity. Supplemental dietary ornithine promotes long-term fecal shedding of *A. baumannii*. By contrast, supplementation of preferred carbon sources—monosodium glutamate or histidine—abolishes the requirement for ornithine catabolism. Additionally, *A. baumannii* gut carriage is higher in formula-fed human infants, who generally consume higher levels of protein, revealing dietary impacts on *Acinetobacter* colonization. Together, these results reveal that ornithine catabolism facilitates *A. baumannii* colonization, providing a reservoir for pathogen spread.

## In brief

Ren and Clark et al. show that *Acinetobacter baumannii* uses ornithine to compete with the resident microbiota and persist in the gut. Additional evidence demonstrates that amino acid supplementation and differences in diet promote *A. baumannii* gut colonization in mice and humans.

## Graphical Abstract

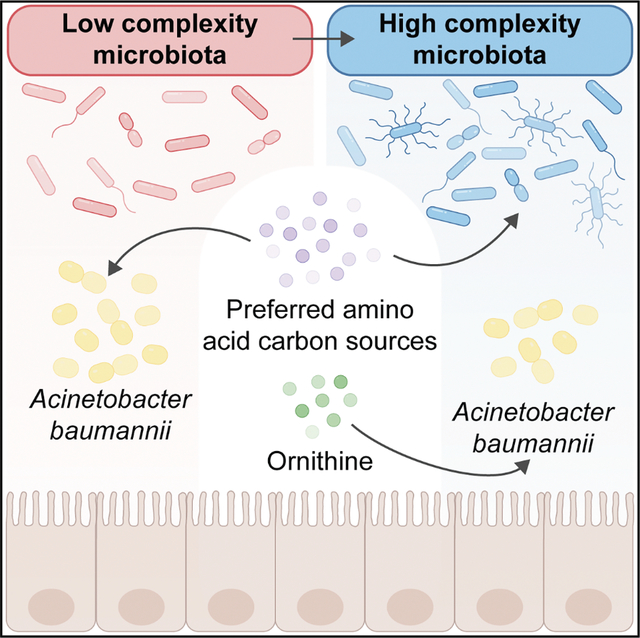

## INTRODUCTION

Antimicrobial resistance (AMR) is a critical threat to human health worldwide. AMR was associated with approximately 5 million deaths in 2021.^1^ Pathogens encoding AMR often persist in hospitals and healthcare settings by asymptomatically colonizing the microbiota of patients and staff.^2,3^ *A. baumannii* is a health-care-associated opportunistic pathogen and was the second leading cause of deaths directly attributable to AMR in 2021.^1,4^ In 2024, the World Health Organization listed carbapenem-resistant *A. baumannii* as the highest-priority target for antibiotic research and development.^5^ *A. baumannii* can colonize any site in the body and asymptomatic colonization is associated with increased risk of infection and mortality in hospitalized patients.^4,6–11^ As early as 1993, the gut was identified as a reservoir for *A. baumannii* in intensive care unit patients, as gut colonization preceded nasopharyngeal colonization and clinical infection.^12^ While the prevalence of gut carriage of *Acinetobacter* species is low in non-hospitalized populations (<1% of people),^13^ gut colonization with *A. baumannii* can be detected in up to 41% of hospitalized patients.^8,10,12,14–18^

Gut colonization increases the risk of clinical infection and transmission. Prospective culture-based studies showed that fecal colonization with multidrug-resistant (MDR) *A. baumannii* increased the risk of clinical infection 5.2–15.2-fold.^6,9,10^ *A. baumannii* strains that asymptomatically colonize the gut have been isolated from clinical infections in the same patients or after transmission to other patients.^7,19–24^ Moreover, rectal swabs detected carbapenem-resistant *A. baumannii* at a higher rate than axillary or nasal swabs in asymptomatically colonized patients,^16^ emphasizing the gut as a potential reservoir for antimicrobial-resistant strains. In addition to colonizing adults in healthcare settings, *A. baumannii* has been isolated from the neonatal gut and is a threat to infant health.^17,25–27^ Therefore, *A. baumannii* colonizes the gut asymptomatically in healthcare settings, which increases the risk of clinical infections.

Despite evidence for the role of *A. baumannii* gut colonization in patients, few mechanistic studies have explored this phenomenon and the *A. baumannii* niche in the gut ecosystem is unclear. Previous studies demonstrated persistent carriage in mouse models with antibiotics and identified a role for secretory IgA and *A. baumannii* thioredoxin in short-term carriage.^28–31^ However, little is known about the strategies that *A. baumannii* employs to colonize the gut.

For *A. baumannii* to colonize the gut, it must overcome colonization resistance from the resident microbiota, which is often mediated by niche exclusion via competition for nutrients.^32–34^ Thus, *A. baumannii* metabolic processes are likely important determinants for colonization and persistence in the gut.^35–37^ Here, we show that ornithine catabolism contributes to *A. baumannii* gut colonization in a post-antibiotics (post-abx) mouse model. We uncovered a second arginine succinyl transferase (AST) pathway operon only in pathogenic *Acinetobacter* species that encodes a predicted ornithine succinyltransferase (AstO) necessary for catabolizing ornithine. In a post-abx mouse model, *A. baumannii* AstO-dependent ornithine catabolism is required to compete with the resident microbiota to colonize the gut. We report that dietary supplementation of monosodium glutamate or histidine, preferred carbon sources, promotes persistent *A. baumannii* colonization in mice and an association between diet and *A. baumannii* abundance in the gut microbiota of human infants. These findings highlight a metabolic strategy that allows *A. baumannii* to utilize a non-preferred carbon source in the gut environment, which is thought to be a reservoir for pathogen spread in healthcare settings.

## RESULTS

### A partial second AST pathway operon confers ornithine catabolism and is critical for *A. baumannii* gut colonization

**Figure 1.**
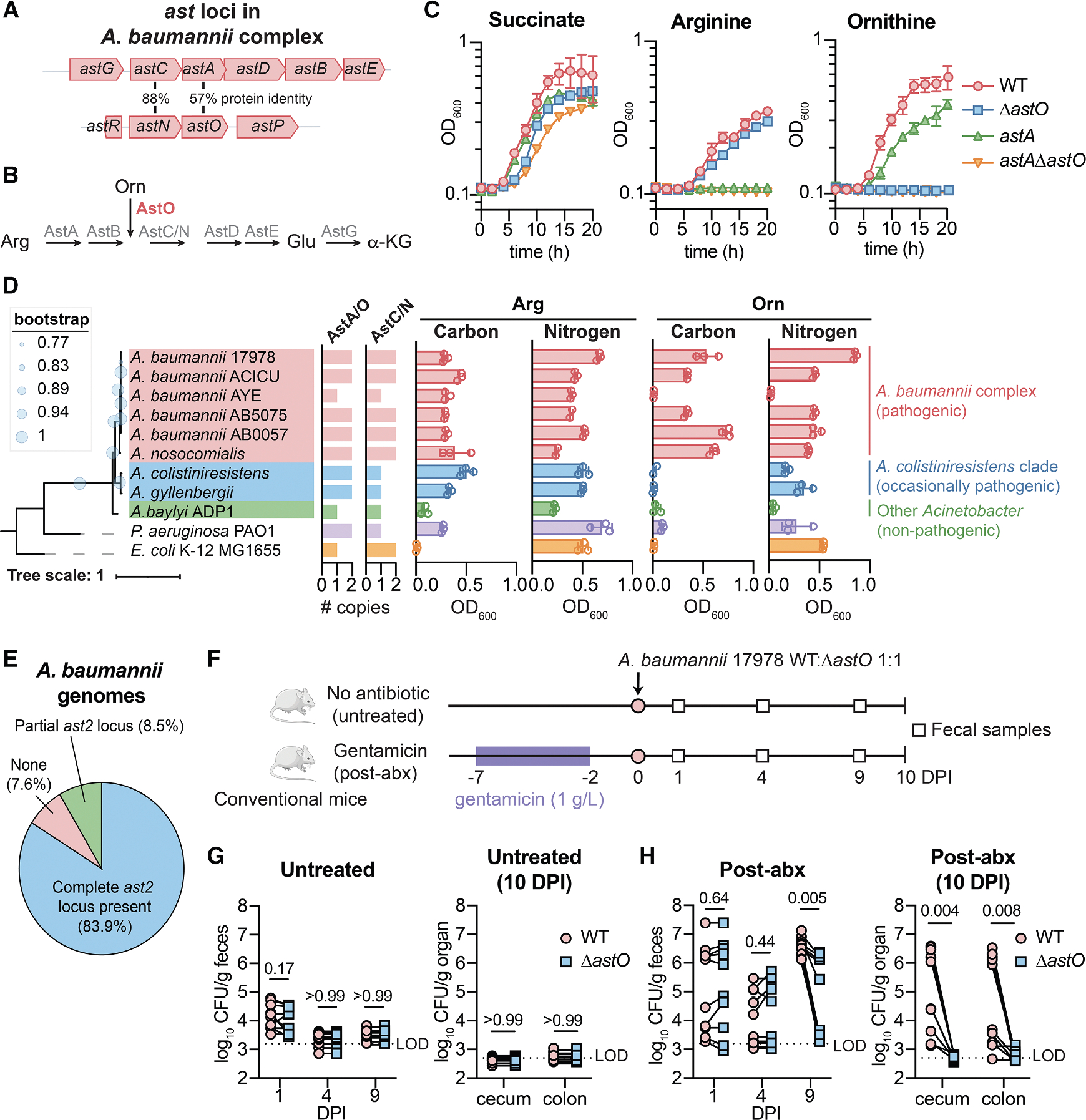
A partial second AST pathway operon confers ornithine catabolism in pathogenic *Acinetobacter* species and contributes to *A. baumannii* gut colonization (A) *ast* loci in the *A. baumannii* complex. (B) Schematic of the predicted arginine succinyltransferase pathway in *A. baumannii*. (C) *A. baumannii* WT, Δ*astO*, *astA*^L125A H229A^ and *astA*^L125A H229A^ Δ*astO* strains were grown in M9 minimal media with the indicated sole carbon source. Growth was monitored by optical density at 600 nm (OD_600_) (*n* = 3, mean ± SD, experiments were repeated at least six times with similar results). (D) Phylogenetic species tree with inferred copy numbers of AstA/O and AstC/N (tree scale in amino acid substitutions). Max OD_600_ of cultures grown with arginine or ornithine as the sole carbon or nitrogen source in M9 medium with succinate (glucose for *E. coli*) or NH_4_ as controls. Growth was over 20 h (*n* = 3, mean ± SD, experiments were performed at least twice with similar results). (E) Prevalence of the *ast2* operon in genomes of *A. baumannii* isolates. (F) Experimental design of post-abx *A. baumannii* gut colonization model. (G–H) *A. baumannii* CFU from fecal samples and in the cecum and colon (*n* = 10 mice combined from 2 independent experiments; *p* by Wilcoxon test with Holm-Sidak’s multiple comparisons). Lines connect CFU of strains enumerated from the same mouse. AST, arginine succinyltransferase; Orn, ornithine; α-KG, α-ketoglutarate; OD_600_, optical density at 600 nm; CFU, colony-forming units; DPI, days post inoculation; LOD, approximate limit of detection. See also Figures S1 and S2 and Table S1.

The molecular mechanisms that govern how *A. baumannii* colonizes the gut are largely unknown. Because competition for nutrients is central to pathogen colonization of the gut, we predicted that *A. baumannii* and related pathogenic *Acinetobacter* species may have evolved additional metabolic capacities to colonize the gut. We identified a partial second AST pathway operon (*astNOP*) in pathogenic *Acinetobacter* species that is absent in non-pathogenic *Acinetobacter* species (Figures 1A and S1A). The AST pathway is typically encoded in a single polycistronic operon *astCADBE* and degrades L-arginine to L-glutamate in *Escherichia coli*, *Pseudomonas aeruginosa*, and *Klebsiella aerogenes*.^38–40^ The *A. baumannii* AST pathway has been shown to be required for one mechanism of polymyxin resistance and lung infection in neutropenic mice.^41,42^

In *A. baumannii* ATCC 17978, the second *ast* locus encodes homologs of AstC (AstN for N_2_-succinylornithine transaminase), AstA (AstO for ornithine succinyltransferase), a putative amino acid permease (AstP for predicted permease), and a divergently transcribed predicted Lrp/AsnC-family regulator (AstR for predicted regulator) (Figures 1A and S1A). In *E. coli* and *P. aeruginosa*, AstA can utilize arginine and ornithine as a substrate to enter the AST pathway.^38,39^ Therefore, we hypothesized that AstO is an ornithine succinyltransferase that allows *A. baumannii* to utilize ornithine (Figure 1B). To test this, *A. baumannii* ATCC 17978 *astA* and *astO* mutant strains were constructed. Multiple attempts to delete *astA* led to polar effects on downstream genes in the operon; therefore, an *astA*^L125A,H229A^ mutant (*astA* mutant) was constructed with substitutions at predicted catalytic residues.^43^ All strains grew with succinate as the sole carbon source (Figure 1C). The wild-type (WT) strain grew with either arginine or ornithine as the sole carbon source (Figure 1C). The *astA* mutant grew with ornithine but not arginine as the sole carbon source (Figure 1C). The Δ*astO*:: Kn (Δ*astO*) mutant grew with arginine but not ornithine as the sole carbon source (Figure 1C). The Δ*astO* mutant defect in growth with ornithine was complemented by *astO* expression in *trans* (Figure S1B). These data suggest *A. baumannii* uses AstA to catabolize arginine and AstO to catabolize ornithine. The double-mutant *astA* Δ*astO* could not utilize either arginine or ornithine (Figure 1C), demonstrating that the AST pathway is the only arginine/ornithine catabolic pathway in *A. baumannii* under these conditions.

We next investigated the phylogeny and conservation of *astO* and ornithine catabolism among *Acinetobacter* species and *A. baumannii* isolates. The *astCADBE* operon is ubiquitously encoded in the *Acinetobacter* genus. The *A. baumannii* complex includes all significant pathogens and encodes *astNOP* with divergent *astR* locus (pink, Figures 1A, 1D, and S1A). Another group that includes occasional pathogens encodes only *astO* near an unrelated LysR-type regulator (*A. colistiniresistens* clade, blue, Figures 1D and S1A).^44^ *A. colistiniresistens astO* can partially complement the Δ*astO* defect in growth on ornithine as the sole carbon source, showing that it is an ornithine succinyltransferase (Figure S1B). Other clades of *Acinetobacter* that are non-pathogenic encode only the *astGCADBE* operon (other *Acinetobacter*, green, Figures 1D and S1A). Because AstO activity in pathogenic *Acinetobacter* clades is similar to that of other bacteria such as *E. coli* and *P. aeruginosa*, we considered the hypothesis that an ancestor of *A. baumannii* acquired the *astO* from another organism via horizontal gene transfer (HGT). Phylogenetic analysis showed that AstA and AstO proteins in the pathogenic *A. baumannii* complex and *A. colistiniresistens* clades were more closely related to each other than to AstA encoded by *Yersinia enterolitica*, *P. aeruginosa*, or *E. coli* (Figure S1C). This suggests that *A. baumannii astO* was acquired via HGT from a closely related species or from a partial duplication after the divergence of pathogenic *Acinetobacter* clades.

To test whether AstO was associated with the ability to use ornithine as the sole carbon and/or nitrogen source in pathogenic *Acinetobacter* species, we quantified AstC/N and AstA/O homologs among select *A. baumannii* strains and *Acinetobacter* species. Encoding only one homolog indicated the presence of only the *astGCADBE* operon, while encoding two homologs indicated presence of a second *ast* locus (Figure 1D). We next measured whether selected strains and species could use arginine and/or ornithine as the sole carbon and energy source in minimal media. Encoding AstO (i.e., encoding two homologs of AstA/O) correlated to whether the *Acinetobacter* species could use ornithine as the sole carbon and/or nitrogen source (Figure 1D). Together, these data demonstrate an association between encoding AstO and ornithine utilization and suggest that the *ast2* locus is associated with pathogenic *Acinetobacter* species.

To determine whether the second *ast* locus is conserved among *A. baumannii* isolates, the prevalence of *astR* and *astNOP* was assessed among our previously described set of 229 *A. baumannii* genomes deduplicated to remove clonal lineages (e.g., outbreak sequencing).^45^ The complete *ast2* locus was present in 83.9% of *A. baumannii* isolates, 8.5% of *A. baumannii* isolates encoded a partial *ast2* locus, and 7.6% of *A. baumannii* isolates lack the *ast2* locus (Figures 1E and S1D; Table S1). Therefore, the *ast2* locus is conserved in most *A. baumannii* isolates, suggesting that AstO-dependent ornithine catabolism confers a fitness advantage to *A. baumannii*.

Ornithine has emerged as key nutrient for gut colonization by the antimicrobial-resistant pathogen *Clostridioides difficile*.^46–49^ To test whether *A. baumannii* uses ornithine catabolism for gut colonization, we developed a post-abx mouse model of *A. baumannii* gut colonization due to the association between previous antibiotic treatment and *A. baumannii* gut colonization in humans.^9,20,23,50,51^ Female mice were administered 1 g/L gentamicin in the drinking water for 5 days or a vehicle treatment (untreated; Figure 1F). Gentamicin was selected because it is a broad-spectrum antibiotic widely used in hospitalized patients.^52–54^ After 2 days of recovery, mice were orogastrically inoculated with 10^9^ CFU of a 1:1 mixture of *A. baumannii* 17978 WT:Δ*astO* (Figure 1F). Consistent with modeling asymptomatic colonization, there were no clinical signs of infection such as hunching or weight loss (Figure S1E). There was no significant difference in the burdens of *A. baumannii* between WT and Δ*astO* in the feces at 1 day post infection (DPI) in the post-abx group or untreated group (Figures 1G and 1H). *A. baumannii* was below the limit of detection (LOD) at 4 DPI in untreated mice, suggesting colonization resistance by the intact resident microbiota (Figure 1G). However, there was *A. baumannii* gut colonization to 10 DPI in the post-abx group (Figure 1H). WT *A. baumannii* significantly outcompeted the *ΔastO* mutant by 9 DPI (Figure 1H). These data suggest that *A. baumannii* ornithine catabolism is required for persistent gut colonization in this post-abx model. At 10 DPI, WT *A. baumannii* CFU were significantly higher than the Δ*astO* mutant strain in the cecum and colon (Figure 1H). Of the 4/10 mice that had detectable CFU in the small intestine, 4/4 had higher WT CFU compared with Δ*astO* in the small intestine at 10 DPI (Figure S1F). There were no *A. baumannii* CFU detected in the spleen at 10 DPI, suggesting that *A. baumannii* did not disseminate to other organs (Figure S1G). There were no differences in transcript abundance of genes encoding pro-inflammatory proteins following antibiotic treatment or *A. baumannii* inoculation, consistent with asymptomatic colonization (Figures S1H–S1I). AstO-dependent *A. baumannii* gut colonization was observed in co-inoculated males and mono-inoculated females, demonstrating that this phenomenon did not depend on sex or co-inoculation (Figures S2A and S2B). Furthermore, AstO-dependent *A. baumannii* gut colonization was observed after pre-treatment with broad-spectrum antibiotics streptomycin or ampicillin, showing that it is not specific to gentamicin (Figures S2C–S2E). However, the temporal dynamics varied among antibiotics and *A. baumannii* did not colonize after treatment with vancomycin, a narrow-spectrum antibiotic primarily targeting gram-positive bacteria (Figures S2C and S2F). Finally, Δ*astO* mutants had no defect in mouse models of lung infection or bloodstream infection (Figures S2G and S2H), the most common types of *A. baumannii* infections.^55,56^ Collectively, these findings suggest that AstO-dependent ornithine catabolism is conserved in pathogenic *Acinetobacter* species and is important for *A. baumannii* persistence in the gut.

### Supplemental dietary ornithine promotes long-term *A. baumannii* gut colonization in mice

**Figure 2.**
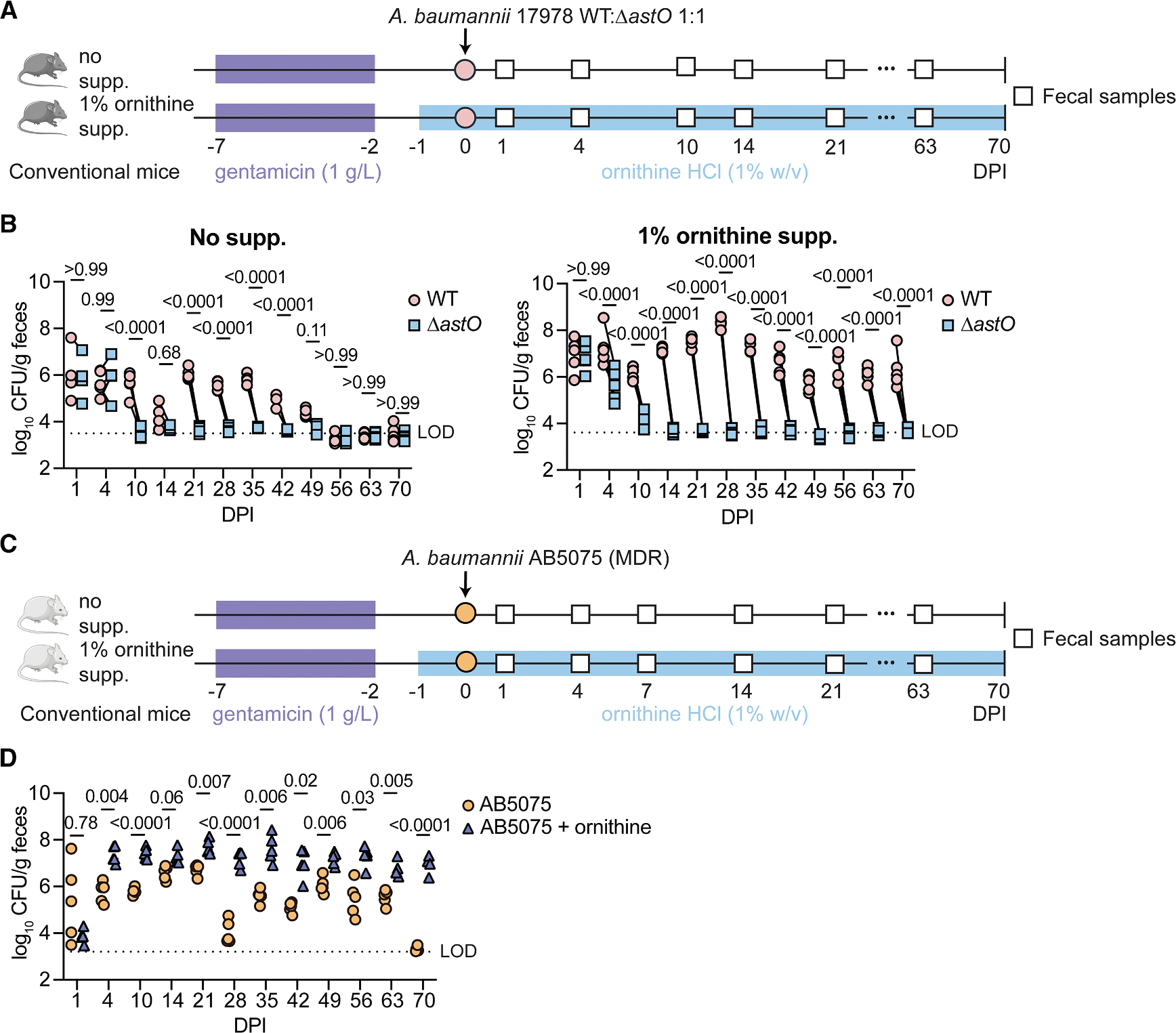
Supplemental dietary ornithine promotes long-term *A. baumannii* gut colonization and fecal shedding in mice (A) Experimental design for ornithine supplementation and *A. baumannii* 17978 inoculation. (B) *A. baumannii* 17978 CFU from fecal samples. (*n* = 5, *p* by two-way ANOVA with Sidak’s multiple comparisons; experiments were repeated twice with similar results.) (C) Experimental design for ornithine supplementation and *A. baumannii* AB5075 inoculation. (D) *A. baumannii* AB5075 CFU from fecal samples (*n* = 5, *p* by two-way ANOVA with Sidak’s multiple comparisons). Lines connect CFU of both strains enumerated from the same mouse. CFU, colony-forming units; DPI, days post inoculation; LOD, approximate limit of detection. See also Figure S3.

Based on the observation that ornithine catabolism is required for *A. baumannii* to persistently colonize the gut, we hypothesized that dietary supplementation of ornithine would promote long-term *A. baumannii* gut colonization. Mice were inoculated with 1:1 WT:Δ*astO A. baumannii* 17978. One group was supplemented with dietary ornithine HCl (1% w/v) in the drinking water 1 day prior to inoculation, and both groups were monitored for 10 weeks (Figure 2A). This concentration was previously shown to promote asymptomatic *C. difficile* colonization.^47^ Without supplemental ornithine, *A. baumannii* 17978 WT outcompeted Δ*astO* from 10–42 DPI (Figure 2B). By contrast, in mice given supplemental dietary ornithine in the drinking water, *A. baumannii* 17978 WT colonized the gut to at least 70 DPI, while the Δ*astO* mutant was at or below the LOD from 14 DPI (Figures 2B, S3A, and S3B). *A. baumannii* 17978 was not detected in the spleens of either group at 70 DPI (Figure S3C). Similar phenotypes were observed in a replicate experiment with a different diet, where WT outcompeted Δ*astO* at least 70 days with or without ornithine supplementation (Figure S3D). Together, these data suggest that *A. baumannii* AstO-dependent ornithine catabolism is required for long-term colonization and that dietary ornithine supplementation promotes persistent *A. baumannii* colonization.

We next tested whether dietary ornithine supplementation similarly promoted gut colonization by *A. baumannii* AB5075, a recent MDR isolate (Figure 2C).^57^ The group receiving dietary supplementation of ornithine in the drinking water had significantly higher *A. baumannii* AB5075 gut colonization than the control group starting at 4 DPI (Figure 2D). *A. baumannii* AB5075 colonized to 63 DPI in the control group and at least 70 DPI in the supplemental ornithine group (Figures 2D, S3E, and S3F). Moreover, *A. baumannii* disseminated to the spleen at 70 DPI when mice inoculated with AB5075 were given supplemental ornithine (Figure S3G). This suggests that supplemental dietary ornithine and/or high bacterial burdens in the gut may promote dissemination of *A. baumannii* AB5075 to the spleen in this model. Together, these data demonstrate that supplemental dietary ornithine promotes persistent gut colonization and may promote dissemination by *A. baumannii*.

### The microbiota determines the fitness advantage from AstO-dependent ornithine catabolism

**Figure 3.**
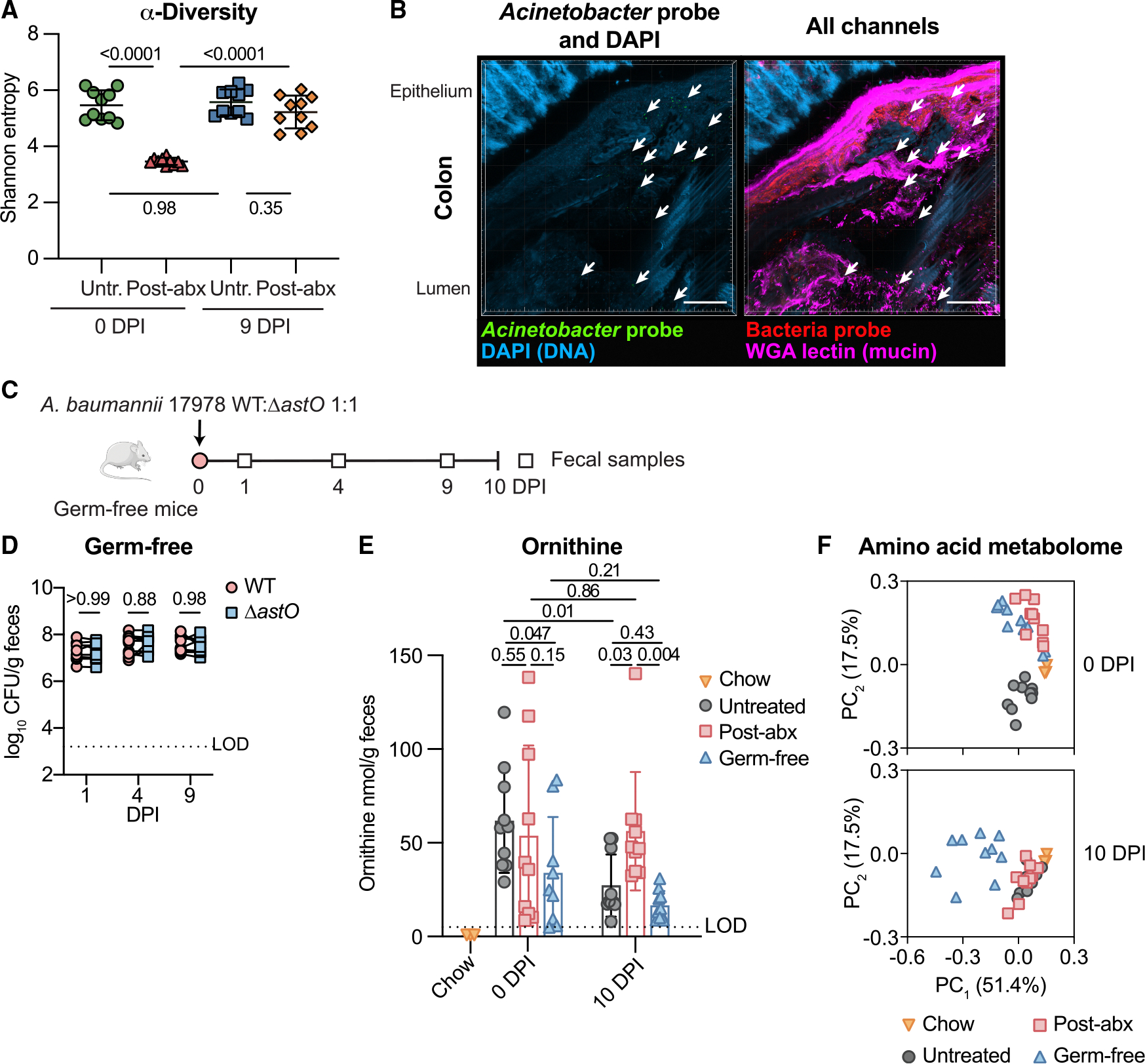
Microbiota diversity associates with *A. baumannii* AstO-dependent ornithine utilization and the gut amino acid metabolome (A) α-Diversity was calculated from 16S rRNA gene sequencing at 0 and 9 DPI of *A. baumannii* WT and Δ*astO* (*n* = 10; data combined from mice in Figures 1G and 1H; mean ± SD are shown; *p* by one-way ANOVA with Sidak’s multiple comparisons). (B) Representative image of *A. baumannii* WT colonization at 10 DPI in the colon, visualized by MiPACT-HCR. Scale bar is 100 μm. Red, pan-bacteria HCR; blue, DAPI; magenta, WGA-lectin; green, anti-*Acinetobacter* HCR. From mice in Figures S4D and S4E. (C) Experimental design with germ-free mice. (D) *A. baumannii* CFU from fecal samples of germ-free mice (*n* = 6 female and *n* = 4 male mice, *p* by two-way ANOVA with Sidak’s multiple comparisons; experiment was repeated three times with similar results). (E) L-ornithine was quantified in chow and fecal samples (chow samples are *n* = 3 [LOD = 1 nmol/g], fecal samples are *n* = 10 mice shown in Figures 1G, 1H, and 3D [LOD = 5 nmol/g], mean ± SD, *p* by two-way ANOVA with Fisher’s least significant difference multiple comparisons only on fecal samples). (F) Principal-component analysis of amino acids in fecal samples at 0 and 10 DPI with chow samples shown in each plot (*n* = 10 mice shown in Figures 1G, 1H, and 3D, chow *n* = 3). Lines connect CFU of both strains enumerated from the same mouse. DPI, days post inoculation; MiPACT-HCR, microbial identification after passive CLARITY technique via hybridization chain reaction; WGA, wheat germ agglutinin; CFU, colony-forming units; LOD, approximate limit of detection; PC, principal component. See also Figure S4 and Table S2.

Since *A. baumannii* colonization relied on microbiota disruption, we next explored whether *A. baumannii* utilizes ornithine to compete with the microbiota. First, the microbiota composition was profiled in fecal samples from the untreated and post-abx mice at 0 and 9 DPI in Figure 1 by 16S rRNA gene sequencing. Microbiota α-diversity, a measure of richness and evenness on a local scale (e.g, in a single mouse), was reduced at 0 DPI in post-abx mice compared with untreated mice but the same in post-abx and untreated mice by 9 DPI (Figure 3A). β-Diversity, a measure of richness and evenness across samples, and relative abundance of amplicon sequence variants (ASVs) were dramatically altered in the post-abx group compared with the untreated group at 0 DPI and largely recovered by 9 DPI (Figures S4A and S4B). There were no differences in total DNA concentrations isolated from fecal samples in both groups at 0 and 9 DPI, suggesting the overall microbial loads were similar (Figure S4C). Together, these data demonstrate that gentamicin treatment dramatically alters the microbiota at the time of inoculation, but that the resident microbiota is largely restored by 9 DPI when *A. baumannii* requires ornithine catabolism.

To determine whether *A. baumannii* is spatially co-localized with the microbiota, *A. baumannii* was visualized *in situ* by microbial identification after passive clear lipid-exchanged acrylamide-hybridized rigid imaging/ immunostaining/ in situ-hybridization-compatible tissue hydrogel (CLARITY) technique with hybridization chain reaction (MiPACT-HCR).^58,59^ Post-gentamicin mice were uninoculated or co-inoculated with *A. baumannii* 17978 WT:Δ*astO*, where WT outcompeted Δ*astO* at 10 DPI (Figures S4D and S4E). At 10 DPI, the colons were harvested for MiPACT-HCR. Imaging revealed that *Acinetobacter* co-localized with bacteria in the lumen of the colon (Figure 3B). The specificity of the HCR probe to *Acinetobacter* was confirmed by imaging cultures of *A. baumannii* or *E. coli* (Figure S4F). The *Acinetobacter* probe displayed low levels of signal in the uninoculated group that also co-localize with the microbiota, which may be endogenous *Acinetobacter* or related genera (Figure S4G). Therefore, *Acinetobacter* in the gut co-localize with other bacteria in the lumen.

Next, we tested whether *A. baumannii* requires AstO-dependent ornithine catabolism in the absence of the microbiota. Germ-free mice were orogastrically inoculated with a 1:1 *A. baumannii* 17978 WT:Δ*astO* (Figure 3C). In the absence of microbiota, *A. baumannii* colonized to high levels, consistent with the idea that the intact resident microbiota confers colonization resistance (Figure 3D). There were no significant differences in colonization by WT and Δ*astO* in germ-free mice throughout the 10-day experiment (Figures 3D and S4H). Thus, ornithine catabolism is not required for *A. baumannii* to colonize mice in the absence of a microbiota.

Ornithine in the gut can be produced by the host or microbiota.^46,60,61^ To determine whether the microbiota was required to produce ornithine, amino acid concentrations were measured from the chow and feces of conventional mice (from Figures 1G and 1H) and germ-free mice (from Figure 3D) at 0 and 10 DPI (Table S2). Ornithine levels in the chow were below the LOD of 1 nmol/g (Figure 3E; Table S2), which was confirmed by manual inspection of chromatograms. Thus, the chow was not a major source of ornithine. In fecal samples, the levels of ornithine were variable and did not correlate with *A. baumannii* abundance or when *A. baumannii* AstO-dependent ornithine catabolism was important for colonization (Figure 3E). Germ-free mice had detectable ornithine in the fecal samples, demonstrating that the host produced ornithine in the absence of a microbiota (Figure 3E). Together, the ornithine abundance in the fecal samples did not easily explain when AstO-dependent ornithine catabolism conferred a competitive advantage to *A. baumannii*. By contrast, the amino acid metabolome of post-abx mice and germ-free mice clustered together and distinctly separate from that of untreated mice at 0 DPI (Figures 3F and S4I). This suggests that the amino acid metabolome differs between groups with varying gut microbiota and correlates to initial colonization resistance. At 10 DPI after the resident microbiota recovered (Figures 3A, S4A, and S4B), the amino acid metabolome of post-abx mice and untreated mice cluster together (Figure 3F). Thus, the amino acid metabolome correlated with gut microbiota recovery and requirement for *A. baumannii* ornithine utilization. Furthermore, these data suggest that *A. baumannii* colonization itself does not disrupt the amino acid metabolome. Collectively, these data are consistent with a model in which antibiotic treatment alters the gut microbiota and amino acid metabolome, disrupting initial colonization resistance to *A. baumannii*. As the resident microbial community recovers, *A. baumannii* must utilize AstO-dependent ornithine catabolism to persist in the gut.

### Glutamate is a preferred carbon source for *A. baumannii* that promotes gut colonization by the Δ*astO* mutant

**Figure 4.**
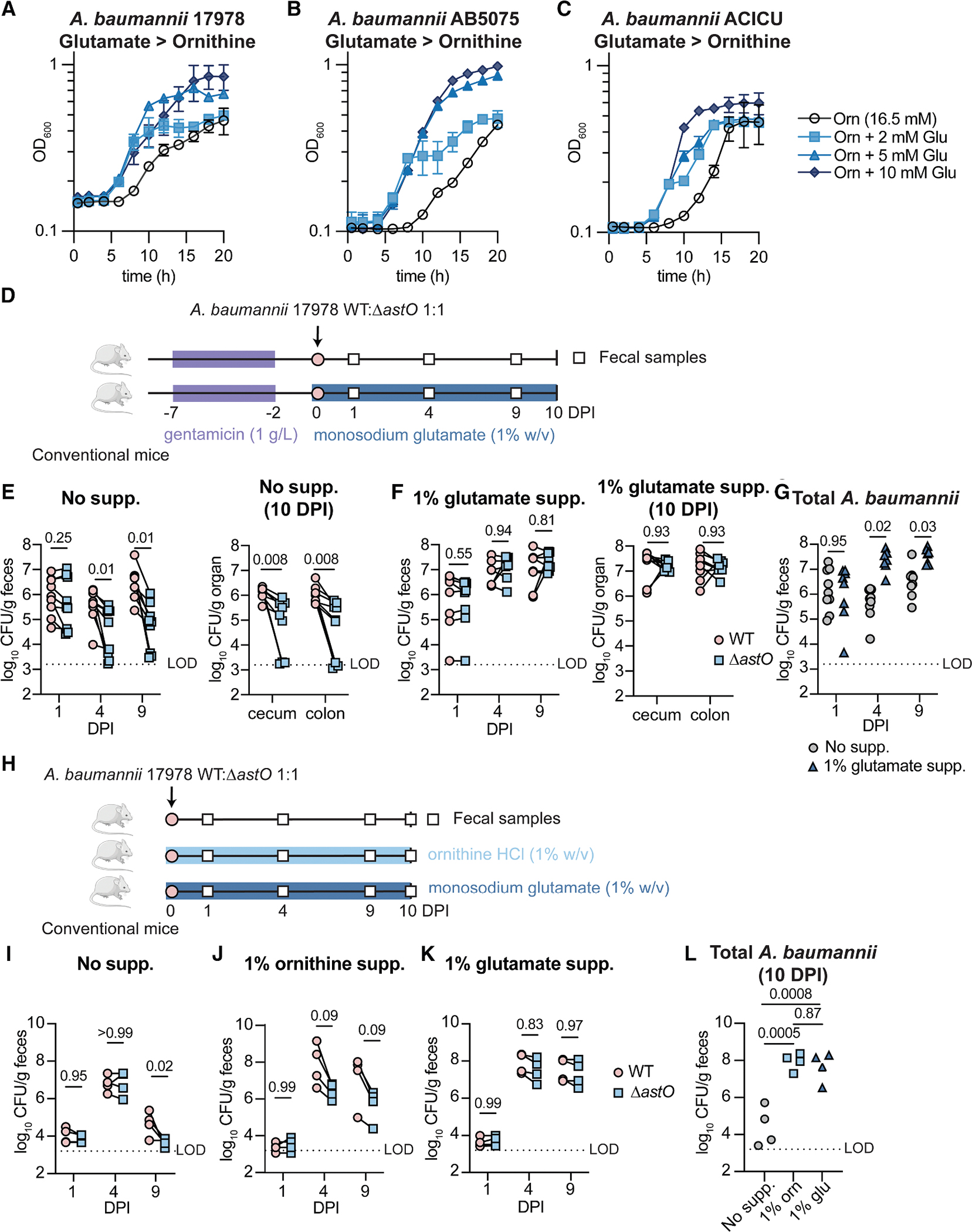
Glutamate is a preferred carbon source for *A. baumannii* that promotes gut colonization by the Δ*astO* mutant (A–C) *A. baumannii* strains were grown in M9 media with the indicated carbon sources. Growth was monitored by OD_600_ (*n* = 3; mean ± SD, experiments were repeated at least four times with similar results). (D) Experimental design for monosodium glutamate supplementation. (E and F) CFU from fecal samples and the cecum and colon (*n* = 8–9 combined from 2 independent experiments, *p* by multiple Wilcoxon tests with Holm-Sidak’s multiple comparisons). (G) Total *A. baumannii* CFU were enumerated from fecal samples (*n* = 8–9, *p* by two-way ANOVA with Sidak’s multiple comparisons). (H) Experimental design for untreated mice supplemented with ornithine or glutamate. (I–K) *A. baumannii* CFU from fecal samples. (*n* = 4; *p* by two-way ANOVA with Sidak’s multiple comparisons.) (L) Total *A. baumannii* CFU from fecal samples (*n* = 4; *p* by one-way ANOVA with Tukey’s multiple comparisons). Lines connect CFU of both strains enumerated from the same mouse. CFU, colony-forming units; DPI, days post inoculation; LOD, approximate limit of detection. See also Figure S5.

We predicted that *A. baumannii* utilizes ornithine when the resident microbiota competes for other carbon sources. To determine whether ornithine is a preferred carbon source for *A. baumannii*, we first sought to determine *A. baumannii* carbon preferences, which had not been described.^36^ *A. baumannii* 17978 was assayed in diauxic growth curves in which the non-preferred carbon source is provided in abundance with limiting concentrations of the preferred carbon source; depletion of the preferred carbon source induces a characteristic delay in growth.^62^ *Acinetobacter* species in general do not use sugars and instead use a wide variety of organic acids and amino acids as carbon sources.^36,63^ Limiting concentrations of ornithine did not induce a delay in *A. baumannii* growth with abundant glutamate or succinate, suggesting ornithine is not a preferred carbon source (Figures S5A and S5B). By contrast, limiting concentrations of other carbon sources (glutamate, succinate, glutamine, or asparagine) induced a delay in growth when added to excess ornithine (Figures 4A and S5C–S5E). Further analysis showed that glutamate, glutamine, histidine, or asparagine are preferred over succinate (Figures S5F–S5I). Finally, ornithine was not preferred over arginine or vice versa (Figures S5J and S5K). The recent MDR clinical isolates *A. baumannii* strain AB5075 and strain ACICU similarly prefer amino acids such as glutamate over ornithine (Figures 4B and 4C), demonstrating conservation of this preference. Together, these data show that *A. baumannii* prefers amino acids such as glutamate, glutamine, histidine, and asparagine over succinate and prefers succinate over arginine and ornithine.

We next hypothesized that dietary supplementation of a preferred carbon source such as glutamate would rescue Δ*astO A. baumannii* colonization. Monosodium glutamate (MSG), a common food component, was supplemented in the drinking water (1% w/v) starting 1 day before inoculation and maintained throughout the 10-day experiment (Figure 4D). Consistent with previous experiments, the Δ*astO* mutant had significantly lower fecal shedding than WT at 4 and 9 DPI and in the cecum and colon at 10 DPI in mice given standard water (Figure 4E). By contrast, mice supplemented with glutamate had higher overall *A. baumannii* burdens and there was no defect for the Δ*astO* strain compared with WT (Figures 4F and 4G).

We further hypothesized that dietary supplementation of amino acid carbon sources would allow *A. baumannii* to overcome colonization resistance without antibiotic disruption of the microbiota. *A. baumannii* colonized mice given ornithine- or glutamate-supplemented water to significantly higher CFU than untreated mice (Figures 4H–4L). Furthermore, the Δ*astO* strain had a significant defect compared with WT in mice supplemented with ornithine (Figure 4J), but no defect in mice supplemented with glutamate (Figure 4K). To determine whether the effect of glutamate supplementation was direct, we attempted to generate an *A. baumannii* mutant defective in glutamate catabolism. However, a double mutant in both annotated glutamate dehydrogenase genes, *astG* and *gdhA*, maintained the ability to catabolize glutamate (Figure S5L). A 99.99% saturating transposon screen in the *astG* Δ*gdhA* mutant background was unable to identify mutants defective in utilizing glutamate, suggesting glutamate dehydrogenase activity is essential or encoded by multiple additional genes. Together, these data show that glutamate is preferred over ornithine and promotes *A. baumannii* colonization directly or indirectly.

### Histidine rescue of *A. baumannii* Δ*astO* gut colonization requires histidine catabolism

**Figure 5.**
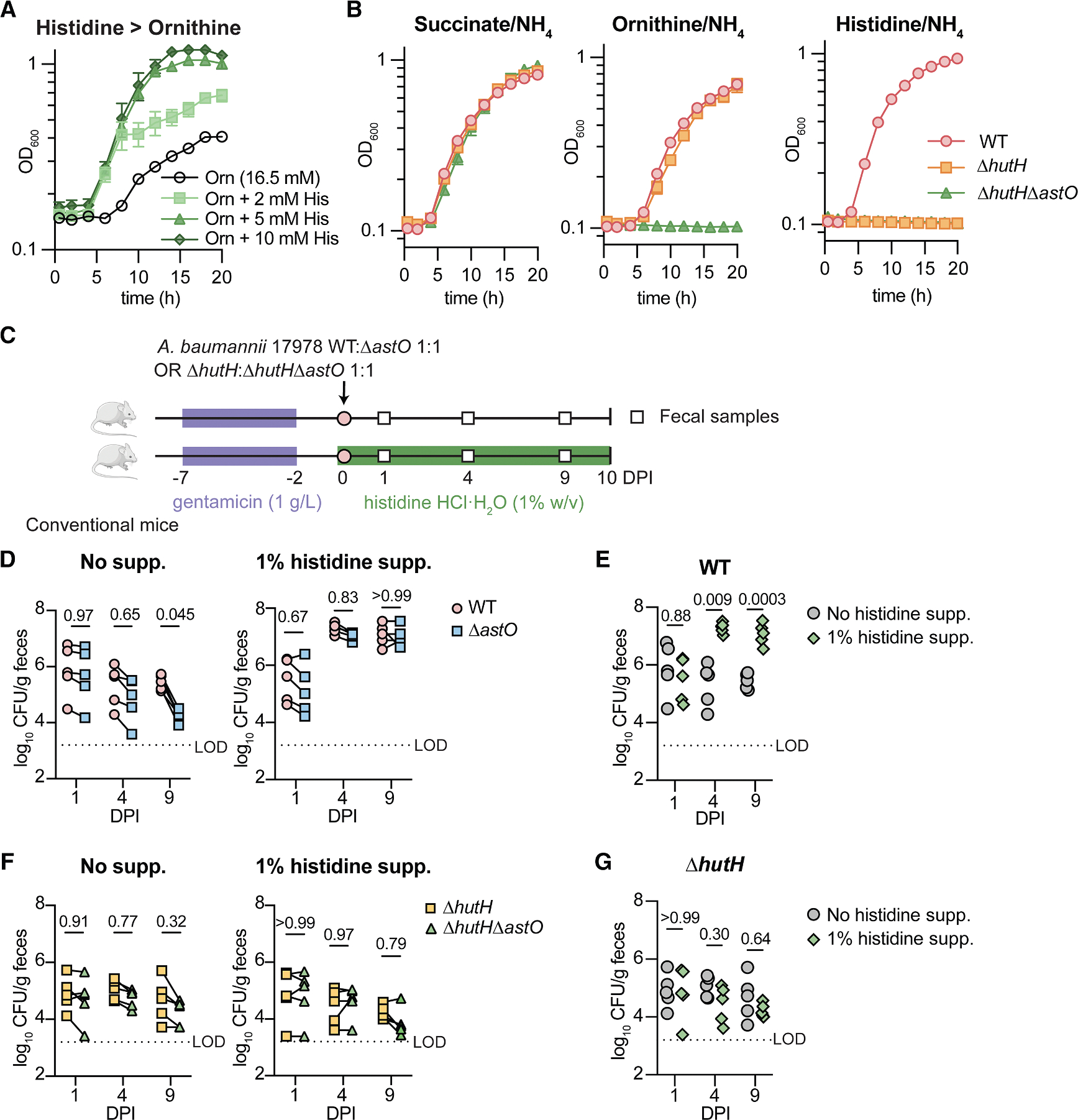
Histidine rescue of *A. baumannii* Δ*astO* gut colonization requires histidine catabolism (A) *A. baumannii* was grown in M9 media with the indicated carbon sources. Growth was monitored by OD_600_ (*n* = 3; mean ± SD, experiment was repeated 4 times with similar results). (B) *A. baumannii* strains were grown in M9 media with the indicated sole carbon source. Growth was monitored by OD_600_ (*n* = 3; mean ± SD, experiments were repeated at least twice with similar results). (C) Experimental design for *hutH* mutant gut colonization with histidine supplementation. (D) Post-abx mice were co-inoculated with *A. baumannii* WT and Δ*astO*. CFU from fecal samples at 1, 4, and 9 DPI (*n* = 5, *p* by two-way ANOVA with Sidak’s multiple comparisons). (E) WT *A. baumannii* CFU from fecal samples with and without histidine supplementation (*n* = 5, *p* by two-way ANOVA with Sidak’s multiple comparisons). (F) Post-abx mice were co-inoculated with *A. baumannii* Δ*hutH* and Δ*hutH*Δ*astO*. CFU from fecal samples (*n* = 5, *p* by two-way ANOVA with Sidak’s multiple comparisons). (G) Δ*hutH* single mutant *A. baumannii* CFU from fecal samples with and without histidine supplementation (*n* = 5, *p* by two-way ANOVA with Sidak’s multiple comparisons). Lines connect CFU of both strains enumerated from the same mouse. OD_600_, optical density at 600 nm; Orn, ornithine; DPI, days post inoculation; LOD, approximate limit of detection. See also Figure S6.

To determine whether preferred amino acids promote Δ*astO* colonization directly or indirectly, we next tested histidine supplementation. The histidine utilization (*hut*) operon is encoded by pathogenic *Acinetobacter* species and the enzyme HutH is required for *A. baumannii* histidine catabolism.^64^ We found that *A. baumannii* preferentially uses histidine over ornithine (Figure 5A). Histidine supplementation also promoted colonization without antibiotics treatment (Figures S6A and S6B). Mutants lacking *hutH* were unable to grow on histidine as the sole carbon source (Figure 5B). Thus, strains in the WT and Δ*hutH* background were compared with test whether histidine supplementation directly promotes *A. baumannii* Δ*astO* persistence in the gut by histidine catabolism (Figure 5C). Similar to glutamate supplementation, histidine supplementation promoted colonization of the WT and Δ*astO* mutant (Figures 5D, 5E, and S6C). By contrast, histidine supplementation did not promote colonization by strains in the Δ*hutH* mutant background (Figures 5F, 5G, S6D, and S6E). This suggests that supplementation of preferred amino acid carbon sources promotes colonization of Δ*astO* strains directly by *A. baumannii* catabolism.

Taken together, these data suggest that *A. baumannii* utilizes preferred carbon sources in the gut when available, such as early colonization in a post-abx model or with supplementation, but *A. baumannii* catabolizes ornithine when the microbiota competes away preferred carbon sources. Furthermore, these findings suggest that dietary amino acids may promote *A. baumannii* colonization in the gut by diminishing colonization resistance from nutrient competition.

### *A. baumannii* abundance in increased in fecal samples from formula-fed healthy human infants

We show that *A. baumannii* can colonize the gut of antibiotic perturbed mice and that supplementation of dietary amino acids promotes *A. baumannii* colonization. To test whether similar factors are associated in humans, we investigated *A. baumannii* abundance in fecal samples from healthy human infants. Infants are born sterile and early life represents a critical window for microbiota colonization and succession. As mentioned above, *A. baumannii* can colonize the gut in neonates and has been associated with an increased risk of bloodstream infection.^17,25–27^ Prior to weaning at around 6 months of age, human infants have relatively similar diets of breastmilk or formula and have a lower complexity microbiome.^65,66^ Notably, ornithine has been detected in breast milk, formula, and infant feces.^67,68^ Infant formula has higher protein levels than breastmilk to protect infants from essential amino acid deficiencies.^69,70^ Fecal metabolomics have shown higher levels metabolites indicative of protein and amino acid catabolism in infants fed formula than breastmilk-fed infants, suggesting proteins and amino acids are dominant nutrient sources for the formula-fed microbiota.^71^ Thus, the human infant gut represents a clinically relevant niche to examine effects of microbiota diversity and diet on *A. baumannii* abundance.

**Figure 6.**
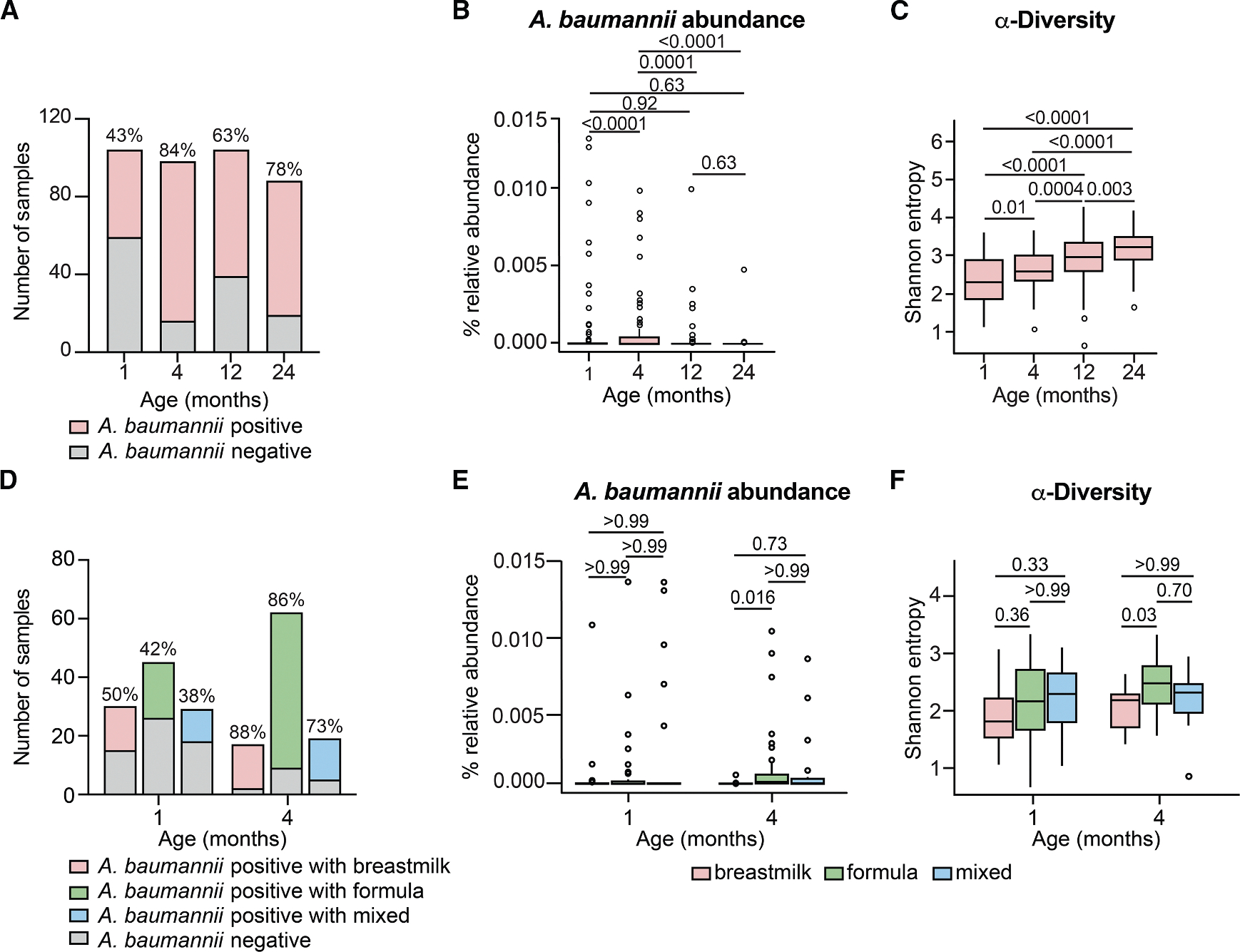
*A. baumannii* abundance in fecal samples from healthy human 4-month-old infants is increased with formula feeding (A–C) Prevalence of *A. baumannii*, relative abundance of *A. baumannii* and microbiota α-diversity in the gut of infants age 1, 4, 12, and 24 months by shotgun metagenomic sequencing (1 month, *n* = 104; 4 months, *n* = 98; 12 months, *n* = 104; 24 months *n* = 88; *p* by Kruskal-Wallis with Dunn’s post hoc test). (D–F) Prevalence of *A. baumannii*, relative abundance of *A. baumannii* and microbiota α-diversity in the gut of infants across feeding types at 1 and 4 months (1 month formula, *n* = 45; 1 month breastmilk, *n* = 30; 1 month mixed, *n* = 29; 4 months formula, *n* = 62; 4 months breastmilk, *n* = 17; 4 months mixed, *n* = 19; *p* by Kruskal-Wallis with Dunn’s post hoc test). Boxplots: center line, median; box limits, upper and lower quartiles; whiskers, 1.5× interquartile range; points, outliers.

Shotgun metagenomic sequencing was analyzed from longitudinal fecal samples from 1- to 24-month-old healthy human infants without exposure to antibiotics. *A. baumannii* was detected in at least 43% of samples in each age group (Figure 6A). The relative abundance of *A. baumannii* increased from 1- to 4-month-old infants and decreased from 4 to 12 and 24 months (Figure 6B). Moreover, the α-diversity of the microbiota increased with age (Figure 6C). This is consistent with the idea that as the microbiota increases in complexity, colonization resistance to *A. baumannii* develops. We next analyzed the pre-weaning infant samples by feeding type. The prevalence of *A. baumannii* was 38%–50% in 1-month-old infants and 73%–88% in 4-month-old infants (Figure 6D). There were no differences in the relative abundance of *A. baumannii* based on feeding type in 1-month-old infants. In 4-month-old infants, when *A. baumannii* relative abundance was highest, there was significantly higher *A. baumannii* relative abundance with formula feeding compared with breastmilk feeding (Figure 6E). Notably, the formula-fed infants had similar or higher α-diversity than breastmilk-fed or mixed feeding type infants (Figure 6F). Thus, the association of formula-feeding with *A. baumannii* abundance is not mediated by reduced diversity of the microbiota. Thus, these data support a model in which *A. baumannii* can colonize a microbiota with reduced diversity and that diet may promote *A. baumannii* gut colonization in humans.

## DISCUSSION

There is a growing appreciation for gut colonization as a reservoir for healthcare-associated pathogens, including *A. baumannii*. Prospective clinical studies have shown that *A. baumannii* gut colonization is a major risk factor for infection and transmission to other patients.^9,21,22^ Here, we report that ornithine catabolism is encoded by pathogenic *Acinetobacter* species and required for *A. baumannii* gut colonization in a post-abx mouse model. Collectively, the data presented here support a model in which *A. baumannii* utilizes ornithine due to competition from the resident microbiota for preferred amino acid carbon sources such as glutamate and histidine. Furthermore, data from mice and humans suggest that dietary amino acids or protein may promote *A. baumannii* gut colonization.

Little is known about the mechanisms that *A. baumannii* uses to colonize the gut. A previous study identified a role for *A. baumannii* thioredoxin A and host secretory immunoglobulin A (IgA) in a short-term colonization model without antibiotics.^31^ By contrast, our study used a post-abx model and observed *A. baumannii* AstO-dependent ornithine catabolism was important for longer-term colonization as the microbiota recovered. Future work could determine whether secretory IgA also promotes long-term colonization. Similar to findings reported here, other studies reported that *A. baumannii* persistently colonized the mouse gut if antibiotic treatment caused microbiota disruption.^28,30^ Additionally, one study showed that 5-fluorouracil, a chemotherapy agent, promoted MDR *A. baumannii* dissemination and mortality, and these effects could be diminished by treatment with probiotic *Bifidobacterium breve*.^30^ The data included here show that ornithine supplementation may also promote systemic dissemination of MDR strain *A. baumannii* AB5075. Together, these findings suggest that environmental factors may contribute to dissemination of MDR *A. baumannii* from the gut to cause invasive infection.

Nutrient competition, including carbon source competition, is an important mechanism by which the gut microbiota limits pathogen colonization.^32–34,72,73^ Amino acid metabolism has been shown to be important for multiple pathogens in the gut and microbiota effects on the host.^46,74–83^ Ornithine specifically is reported to promote non-inflammatory *C. difficile* gut colonization, toxin titers, and spore formation.^47–49^ *C. difficile* is specifically adapted to the gut environment, where it competes with related microbiota members for nutrients.^84–86^ *A. baumannii* is not a specialized gut microbe. Thus, the fact that both *A. baumannii* and *C. difficile* utilize ornithine to colonize the gut support and extend previous findings that ornithine utilization is an exploitable nutrient niche for invading pathogens. Data shown here suggest that *A. baumannii* uses ornithine in the gut when the diverse resident microbiota competes away preferred carbon sources. Although ornithine in the gut can be from the diet, the multiple chows used here had ornithine below the LOD (1 nmol/g). Rather, ornithine was likely produced by the host such as by enteral arginase 2 expression,^60^ and other members of the gut microbiota. Examples include the arginine deiminase pathway in *Lactobacillus* and *Enterococcus* spp.,^46,61^ and arginase in many genera including *Bacillus spp*.^87^ One recent study found that bacteria from the phylum *Bacillota* (formerly *Firmicutes*) are more likely to prefer protein as a carbon source, suggesting potential mediators of colonization resistance to *A. baumannii*.^88^ In our mouse model, multiple *Bacillota* ASVs (e.g., *Clostridia* and *Baccilli*) were depleted at 0 DPI post-abx and recovered by 9 DPI (Figure S4B), suggesting that they may play a role in competitively excluding *A. baumannii* from preferred carbon sources.

Dietary amino acids and protein could play an important role in *A. baumannii* colonization and pathogen colonization in general. Ornithine, glutamate, and histidine are sold commercially as supplements, and MSG is a common food additive. For infants and neonates, formula typically has higher concentrations of protein than breastmilk,^70^ potentially providing more amino acid nutrients for *A. baumannii* to colonize. However, there are multiple differences between formula and breastmilk such as hormones, immunoglobulins, and other antimicrobial effectors that could contribute to *A. baumannii* colonization resistance. Previous studies showed that a high-protein diet fed to conventionally raised mice enhanced *Citrobacter rodentium* colonization^74^ and that low dietary protein protects from *C. difficile* colonization.^78^ Together the findings reported here along with previous studies are consistent with a role for amino acids and dietary protein in promoting gut microbiota invasion by some pathogens.

In conclusion, this study uncovers a metabolic strategy that *A. baumannii* uses to persist in the gut in the face of competition for nutrients by the resident microbiota. The data presented here support a model in which a diverse microbiota outcompetes *A. baumannii* for preferred carbon sources, thus *A. baumannii* catabolizes ornithine to persist in an available nutrient niche. Future research into *A. baumannii* nutritional requirements in the gut and the key impacts of other members of the microbiota will provide a foundation to develop new strategies to decolonize *A. baumannii* and prevent infections. These findings have significant clinical relevance, as gut colonization is thought to be important AMR reservoir in healthcare facilities.

## RESOURCE AVAILABILITY

### Lead contact

Requests for further information and resources should be directed to and will be fulfilled by the lead contact, Lauren D. Palmer (ldpalmer@uic.edu).

### Materials availability

All materials generated in this study are available from the lead contact with appropriate material transfer agreement.

### Data and code availability

- All data are available in the NCBI sequence read archive (SRA) with the following accessions: metagenomic sequencing from healthy human infants, NCBI: PRJNA1145027, PRJNA1106565, PRJNA1042647, and PRJNA1173239; whole-genome sequencing for *A. baumannii* 17978VU *att::*mTn*7* and Δ*astO*::Kn, NCBI: PRJNA1173193; mouse 16S rRNA gene sequencing, NCBI: PRJNA1178140.
- This paper does not report original code.
- Any additional information required to reanalyze the data reported in this paper is available from the lead contact.

## STAR★METHODS

### EXPERIMENTAL MODEL AND SUBJECT DETAILS

#### Ethics statements

##### Mouse studies

All animal experiments have been performed in agreement with NIH guidelines, the Animal Welfare Act, and US federal law. All animal experiments were approved by the Institutional Animal Care and Use Committee in protocol 20–165, 23–119 and 22–192 at University of Illinois at Chicago. All mice were euthanized by methods consistent with American Veterinary Medical Association (AVMA) guidelines.

##### Human studies

Samples were from infants who had never received antibiotics and had no history of health problems at the Children’s Hospital of Philadelphia as part of the Infant Growth and Microbiome (IGraM) Study, a prospective, longitudinal cohort study of pregnant African American women and their infants. The human study protocol was reviewed and approved by the Committee for the Protection of Human Subjects (Internal Review Board) of the Children’s Hospital of Philadelphia, protocol 14–010833. The data used here were consistent with the stated purpose of the research. The Institutional Review Board-approved consent documents included language that allowed participants to indicate whether they would like to have their information included in future research. Subjects may participate in the original research without their information (even if de-identified) being included in future research. The age at which samples were collected were 1 month, 4 months, 12 months, and 24 months, as indicated in the figures. The sample sizes for each analysis are indicated in the figure legends. The cohort included 52 female and 56 male infants.

##### Mouse strains and husbandry

###### Conventional SPF mice.

Five-to-six-week-old female or male Swiss Webster mice were purchased from Charles River Laboratories, except for mice for experiments in Figure 5 which were from Inotiv. Swiss Webster mice were used for all 10-day experiments with *A. baumannii* 17978 inoculation gut colonization experiments and the 10-week experiment with *A. baumannii* AB5075. Mice were fed autoclaved mouse diet 5L79 (PMI Nutrition International 5L79). For Figures 2A and 2B, six-week-old female C57BL/6J mice were purchased from The Jackson Laboratory and fed irradiated LM-485 (Teklad 7912). For Figure S3D, six-week-old female C57BL/6J mice were purchased from the Jackson Laboratory and fed irradiated NIH-31 Modified Open Formula (Teklad 7913). Ornithine in 5L79, LM-485, and NIH-31 Modified Open Formula was confirmed to be <1 nmol/g (Table S2). For the lung infection experiment, mice were 7-week-old male C57BL/6J mice from The Jackson Laboratory fed irradiated LM-485 (Teklad 7912). For the bloodstream infection experiment, mice were 7-week-old female C57BL/6J mice from The Jackson Laboratory fed irradiated LM-485 (Teklad 7912). All mice were randomly assigned to experimental and control groups and were kept in the Biologic Resources Laboratory (BRL) facility at the University of Illinois Chicago. The mice were housed with 14 h:10 h light/dark cycles, 70–76°F and 30%–70% humidity.

###### Germ-free mice.

Germ-free Swiss Webster (Tac:SW) WT mice were purchased from Taconic and bred in the BRL facility at the University of Illinois Chicago in a room with 14 h:10 h light/dark cycles and 70–76°F and 30%–70% humidity. Mice were kept in isolators purchased from Park Bioservices LLC. Mice were fed autoclaved mouse diet 5L79 (PMI Nutrition International 5L79) and autoclaved super Q water *ad libitum* and were 12–16 weeks of age. Germ-free condition was tested at least once a month with aerobic liquid cultures (brain heart infusion, BHI), solid cultures (blood agar plates, Thermo Sci Remel), fungal cultures (Sabouraud slants), anaerobic liquid (BHI) and solid cultures (*Brucella* agar, Thermo Sci Remel) from isolators’ swabs, fecal samples, and fungal traps placed inside the isolators. Fecal samples were also tested with Gram staining and qPCR to detect bacterial DNA.

#### Bacterial strains and culture conditions

*A. baumannii* strains, *E. coli* K12, and *Pseudomonas aeruginosa* PAO1 were grown in LB ((10 g/L tryptone, 5 g/L yeast extract, 10 g/L sodium chloride) or LB agar (10 g/L tryptone, 5 g/L yeast extract, 10 g/L sodium chloride, 15 g/L agar) at 37°C. *A. baylyi*, *A. gyllenbergii* and *A. colistiniresistens* were grown in LB or LB agar at 30°C. Antibiotics were used at the following concentrations: carbenicillin 75 mg/L, kanamycin 40 mg/L, chloramphenicol 15 mg/L. For experiments with mice, the antibiotics used for selective plating were used at the following concentrations: carbenicillin 50 mg/L, kanamycin 40 mg/L, chloramphenicol 5 mg/L.

### METHOD DETAILS

#### Bacterial strain construction

The strains, recombinant DNA, and oligonucleotides used in this study listed in the Key Resource Table. *A. baumannii* 17978 is ATCC 17978VU.^89^ Mutants were generated by allelic exchange with sucrose counterselection using pFLP2.^92^ Approximately 1,000 bp of DNA in both the 5′ and 3′ flanking regions surrounding targeted genes was amplified using *A. baumannii* genomic DNA as a PCR template. Oligonucleotides were purchased from Integrated DNA Technologies. The kanamycin resistance marker was amplified from the vector pKD4.^93^ The PCR products were cloned into the pFLP2 vector using HiFi Assembly (New England Biolabs). The pFLP2 constructs were then introduced into WT *A. baumannii* by electroporating and selecting for Kn^R^; resulting colonies were further screened for Carb^R^ and sucrose^S^. Then the sucrose sensitive and PCR-confirmed merodiploids were plated to LB agar containing 10% sucrose to select for loss of the plasmid. Resulting colonies were screened for Kn^R^ and Carb^S^. The Δ*astO*::Kn strain was confirmed by PCR and whole genome sequencing. For construction of the site mutations *astA*^*H229A*,*L125A*^ and *astG*^*K76A,D147A*^, 1,000 bp of DNA in both the 5′ and 3′ flanking regions surrounding the targeted nucleotides was amplified by PCR. These PCR products were cloned into the pFLP2 vector using HiFi Assembly. After sequence confirmation, the resulting plasmids were introduced into WT by electroporating, selecting for Carb^R^, and screening for sucrose^S^. Then the sucrose sensitive clones were plated onto agar containing 10% sucrose to select for loss of the plasmid. The resulting colonies were screened for Carb^S^ by patching and for the site mutation by amplifying the region by PCR and sequencing. For construction of the double-mutant strain *astA*^*H229*^,^*L125A*^Δ*astO*::Kn, the pFLP2-*astO*::Kn plasmid was introduced into *astA*^*H229A, L125A*^ by electroporating and selecting as described above. For construction of the double glutamate dehydrogenase mutant, *astG*^*K76A*^, ^*D147A*^Δ*gdhA*::Kn, the pFLP2-*gdhA*::Kn plasmid was introduced into the *astG*^*K76A, D147A*^ strain.

The markerless *astG*^*K76A, D147A*^Δ*gdhA::FRT*, and Δ*hutH::FRT* strains were constructed as previously described by electroporating the Kn^R^ parental strains with pAT03, inducing the FLP recombinase to remove the kanamycin resistance cassette, and screening for Kn^S^ Carb^S^ colonies that lost the plasmid.^95^ The Δ*hutH*Δ*astO*::Kn mutant was constructed by introducing the pFLP2-*astO*::Kn plasmid into Δ*hutH::FRT*. All strains were confirmed by multiple PCR.

For the *astO* complementation vector, open reading frames (ORFs) were amplified by PCR and cloned into pWH1266 vector with a constitutive *A. baumannii rpsA* promoter (*rpsAp*) by Hifi Assembly.^97,98^ The resulting plasmid was introduced into *A. baumannii* Δ*astO* by electroporating and selecting for Carb^R^ to generate the complementation strain. To construct the WT and Δ*hutH A. baumannii* strains marked with *att::*mTn*7*, the pKNOCK-mTn*7* was transformed into *A. baumannii* WT and Δ*hutH*::*FRT* by a four parental conjugal mating strategy as described previously.^94^

#### Bacterial growth curves

*A. baumannii* strains, *A. nosocomialis*, *P. aeruginosa*, and *E. coli* were grown in 3 mL LB at 37°C overnight with shaking at 180 rpm. Cultures were diluted 1/100 into PBS, then further diluted 1/100 into 99 μL carbon-free nitrogen-free M9 minimal medium with 0.1X trace metal solution^103^ and the indicated carbon source (16.5 mM) and nitrogen source (18.6 mM, ammonium chloride unless otherwise specified) in a flat bottom 96-well plate (Fisher) and covered with Breathe-Easy sealing membrane. Growth was monitored in a BioTek Synergy 2 plate reader or Epoch 2 plate reader by optical density at 600 nm (OD_600_) at 37°C with shaking. *A. baylyi*, *A. gyllenbergii*, and *A. colistiniresistens* were cultured as described but at 30°C.

#### *ast2* locus prevalence in *A. baumannii* strains

Protein sequence identity was determined with EMBOSS needle.^104^ The prevalence of the *ast2* operon was assessed in the previously described set of deduplicated *A. baumannii* genomes.^45^ Briefly, 7,431 genome assemblies were downloaded from NCBI. Genomes with contig N50 scores in the lowest 20% were removed, reducing the dataset to 5,945 genomes. Due to the presence of many closely related genomes, a deduplication process was conducted using Average Nucleotide Identity (ANI) values, estimated via Mash 2.34.^105^ A custom Python script iterated through the genomes, comparing their Mash distances, and discarded genomes with lower N50 scores if they were too similar (based on a threshold distance of 0.006). Additionally, ten genomes identified as outliers were excluded. The code used is previously published and available at https://github.com/JonWinkelman/genome_deduplication.^45^ This resulted in 229 proteomes from filtered and deduplicated *Acinetobacter* genomes (Table S1), with four outgroup species (*A. nosocomialis, A. colistiniresistens*, *A. gyllenbergii*, and *A. baylyi*) that were used to root the species tree. Orthofinder generated hierarchical orthologous groups (HOGs) for each internal node of the species tree, representing proteins descended from a common ancestral gene. HOGs linked to the last common ancestor of all *A. baumannii* species were used. Details on analysis and visualization are available at https://github.com/JonWinkelman/dash_app_Acinetobacters/tree/main.

#### Phylogenetic trees

Trees were inferred with OrthoFinder version 2.5.5^99,106^ or RAxML.^107^ When inferring gene/protein trees, protein alignments for RAxML were produced with muscle 5.1.^108^ The RAxML command was configured to perform phylogenetic analysis using the PROTGAMMAAUTO model, which automatically selects the best-fitting protein substitution model combined with the Gamma model of rate heterogeneity. Rapid Bootstrap analysis and searches for the best-scoring Maximum Likelihood tree were performed in a single run. Random number seeds were provided for both the rapid bootstrap analysis (-x 123) and the parsimony inference step (-p 256). The number of bootstrap replicates was determined automatically using the autoMRE option, which stops generating replicates once a sufficient number have been produced to yield reliable support values.

#### Mouse experiments

Purchased mice were first acclimated in the facility for one week. Conventional mice given antibiotics were then treated with 1 g/L gentamicin, 5 g/L streptomycin, 0.5 g/L ampicillin, or 0.5 g/L vancomycin in the drinking water for 5 days (day −7 to −2); antibiotic water was replaced every 2–3 days; on day −2, all mice were given normal drinking water. For amino acid supplementation, mice received ornithine hydrochloride (1% w/v), monosodium glutamate monohydrate (1% w/v), or histidine monohydrochloride monohydrate (1% w/v, adjusted pH to neutral) in the drinking water from day −1 before inoculation with *A. baumannii* until the end of the experiment. *A. baumannii* 17978 strains were grown in 10 mL LB at 37°C with shaking for 16 h. *A. baumannii* AB5075 was incubated in 10 mL LB at room temperature without shaking overnight, then incubated at 37°C with shaking for 3.5 h. Subsequently, the bacteria were centrifuged at 4°C, 4000 × *g* for 7 min, washed in PBS twice, and resuspended in PBS to 1 × 10^10^ colony-forming units (CFU)/mL. On day 0, mice were orally gavaged with 0.1 mL (1 × 10^9^ CFU) of the indicated strains and placed in a clean cage. Cages were changed at least weekly. For *A. baumannii* CFU enumeration, fecal samples were weighed, resuspended in 1 mL PBS, serially diluted, and plated to LB plates supplemented with appropriate antibiotics. CFU were enumerated on LB agar with 5 mg/L chloramphenicol and 50 mg/L carbenicillin (*A. baumannii* 17978 WT *att*::mTn*7* or *A. baumannii* 17978 Δ*hutH att*::mTn*7*), with 5 mg/L chloramphenicol and 40 mg/L kanamycin (*A. baumannii* 17978 Δ*astO*::Kn or *A. baumannii* 17978 Δ*hutH*Δ*astO*::Kn), with 5 mg/L chloramphenicol (*A. baumannii* 17978 WT), or with 15 mg/L chloramphenicol (*A. baumannii* AB5075 WT). Fecal samples were collected at 0 DPI and plated to confirm that no endogenous bacteria were resistant to plates selective for *A. baumannii* strains. Mice were euthanized with CO_2_. Cecum and colon were collected in 1 mL PBS and the spleen was collected in 0.7 mL PBS and homogenized by Bullet Blender with stainless steel beads (NextAdvance). The serial dilutions of homogenates were plated on LB agar with appropriate antibiotics. For the germ-free mouse experiment, 10^7^ and 10^9^ *A. baumannii* inocula were tested and the data were combined. For the histidine supplementation experiments, WT and Δ*hutH* strains were confirmed from CFU plating by PCR.

For lung infection, *A. baumannii* 17978 WT and Δ*astO* mutant strains were grown overnight in 3 mL LB with shaking at 180 rpm at 37°C for 16 hours. The overnight cultures were then subcultured 1:100 into 10 mL LB and grown for 3.5 h. *A. baumannii* were harvested by centrifugation, washed twice in PBS, and resuspended in PBS at 1 × 10^10^ CFU/mL. Mice were anesthetized with intraperitoneal injection of ketamine/xylazine diluted in PBS. Mice were then inoculated intranasally with 1:1 ratio of *A. baumannii* WT and Δ*astO* at approximately 3 × 10^8^ CFU in 30 μL. At 1 DPI, mice were euthanized with CO_2_ and organs (lungs, spleen, liver, heart, kidneys and nasal cavity) were collected. Organs were homogenized in PBS using a NextAdvance Bullet Blender tissue homogenizer on setting 8. CFU were enumerated on LB agar (total *A. baumannii* 17978), and LB agar with 40 mg/L kanamycin (Δ*astO*::Kn); WT CFU were determined by subtraction.

For retroorbital infection, *A. baumannii* 17978 WT and Δ*astA*Δ*astO* double-mutant strains were prepared as described for lung infections. Mice were anesthetized by isoflurane and inoculated retro-orbitally with a 1:1 ratio of *A. baumannii* 17978 WT and Δ*astA*Δ*astO* at approximately 5 × 10^8^ CFU in 50 μL. At 1 DPI, mice were euthanized, organs (lungs, spleen, liver, heart, and kidneys) were collected, and CFU were enumerated as described for lung infections.

#### RNA extraction and cytokine qRT-PCR

Cecal RNA extraction and qRT-PCR were performed as previously described.^109^ Cecum tissue collected from mice 10 DPI were homogenized by mortar and pestle using liquid nitrogen. The homogenate was transferred to 1 mL of Tri-Reagent (Molecular Research Center) for RNA extraction. RNA was extracted with 0.1 mL of 1-bromo-3-chloropropane, centrifuged, and the upper phase was precipitated with 0.5 mL isopropanol. After centrifugation, pellets were washed twice with 1 mL of 75% ethanol in RNase-free water. The RNA pellet was then resuspended in RNase-free water. RNA was treated with DNase using the Turbo DNA-free kit (Invitrogen). Reverse transcription was performed with the High-Capacity cDNA Reverse Transcription Kit (Applied Biosystems). 500 ng of RNA was used for the reverse transcription reaction. The reverse transcription cycle consisted of 10 minutes at 25°C followed by 120 minutes at 37°C and 5 minutes at 85°C. qRT-PCR was performed using the Fast SYBR Green Master Mix (Applied Biosystems) on the Viia7 Real-time PCR system at the Genome Research core at the University of Illinois Chicago. The qRT-PCR reaction cycle consisted of 20 seconds at 95°C followed by 40 cycles of 3 seconds at 95°C and 30 seconds at 60°C. Reactions were performed in duplicate.

#### MiPACT-HCR imaging

Mice were mono-inoculated with either WT or Δ*astO A. baumannii* 17978 as described above. After euthanasia, the whole colon was fixed in methacrylate (10% acetic acid, 30% chloroform, and 60% methanol). *In vitro* bacterial cultures of *A. baumannii* and *E. coli* were grown in 5 mL LB at 37°C with shaking for 16 h and then 2% (v/v) paraformaldehyde was added. The bacteria were fixed by rocking at 4°C overnight. The cells were then washed with PBS and normalized to an OD_600_ of 1.0 in PBS.

The fixed bacterial cells or organ were then embedded, sectioned and stained as previously described.^59^ Briefly, fixed bacterial cells were embedded once in B4P1 (bis-acrylamide/paraformaldehyde mixture). Colons were embedded twice in B4P1 to ensure retention of lumen contents during clearing. After clearing, the samples were stained with DAPI, and lectin (wheat germ agglutinin [WGA]) conjugated to AlexaFluor-647 (Thermo Scientific). Hybridization chain reaction (HCR) was performed with an *Acinetobacter*-specific FISH probe (Aci-16s 729)^110^ with a B4 overhang and amplified by fluorescent DNA hairpins conjugated to Alexa Fluor-488 (Molecular Instruments) and a pan-bacteria FISH probe (EUB338)^111^ with a B1 overhang amplified by fluorescent DNA hairpins conjugated to Alexa Fluor-647 (Molecular Instruments). Imaging was performed using a Zeiss LSM 780 confocal microscope or a Zeiss LSM 880 confocal microscope with a Plan-Apochromat 10×/0.45-numerical aperture M27 objective for fixed bacterial cells or a STELLARIS 5 confocal microscope (Leica Microsystems) with a 10x/0.4 numerical aperture objective or a 20x/0.75 numerical aperture objective for colon samples. Scanned images were processed using Imaris imaging software (Bitplane) or the FIJI distribution of ImageJ.

#### Mouse microbiota sequencing and analysis

Mouse fecal samples were collected and immediately stored at −80°C until processing. DNA was extracted using by QIAamp PowerFecal Pro DNA Kit according to the manufacturer’s instructions. Library preparation and sequencing was conducted at the Rush University Genomics and Microbiome Core Facility (GMCF). Microbial community characterization was performed using two-stage 16S rRNA gene amplicon sequencing, similar to methods previously described.^112,113^ Briefly, gDNA was amplified with primers targeting the V1-V3 variable regions of microbial 16S rRNA genes using a mixture of 27F primers and 534R primers. Forward primers were a combination of multiple versions of the 27F primer, as described previously,^114^ and including 27F-YM, 27F-Bor, 27F-Bif, 27F-Chl, and 27F-Ato forward primer variants. All primers contained Fluidigm common sequence linkers (CS1, ACACTGACGACATGGTTCTACA, for forward primers; CS2, TACGGTAGCAGAGACTTGGTCT, for reverse primers). PCR reactions were conducted in 10 μL volumes with repliQa HiFi Tough Mix (QuantaBio). PCR conditions included an initial denaturation at 98°C for 2 minutes, followed by 24 cycles of 98°C for 10 seconds, 56°C for 2 seconds, and 68°C for 1 second. Subsequently, a second PCR reaction was performed using 1 μL of the product from the first stage as the input without purification. The second-stage primers were from the Access Array for Illumina barcoding system (Fluidigm, San Francisco, CA, USA) and contained Illumina sequencing adapters, sample-specific barcodes, and CS1 and CS2 linkers at the 3′ ends of the oligonucleotides. The PCR conditions for these second reactions were an initial denaturation at 98°C for 2 minutes, followed by 8 cycles of 98°C for 10 seconds, 60°C for 1 second, and 68°C for 1 second. After the 2nd stage amplification, samples were pooled approximately equimolarly and sequenced on an Illumina MiSeq sequencer and employing a V3 kit with paired-end 2 × 300 base sequencing reads. Demultiplexing was performed on the sequencing instrument.

The resulting paired-end FASTQ files were merged using the Paired-End read merger (PEAR) algorithm.^115^ Merged data were then quality trimmed (≥ 20) and filtered for sequences ≥ 450 bases. The trimmed and filtered sequences were exported as FASTA and analyzed using nf-core/ampliseq version 2.1.0.^116,117^ Amplicon sequence variants (ASVs) taxonomies were assigned by DADA2 using SILVA-138 database.^118–120^ α-diversity, β-diversity, and relative abundance bar plots were generated with QIIME 2.^121^ Nonmetric MultiDimensional Scaling (NMDS) plot of Jaccard β-diversity was generated with the R package vegan (version 2.6–8).

#### Amino acid metabolomics and quantification

Amino acid metabolomics was performed as previously described.^46,122^ Samples were pre-weighed and homogenized in sterile PBS and then sterilized with 0.22-μm filters. Amino acids were derivatized using the Waters AccQ-Tag Ultra Amino Acid Derivatization Kit (Waters Corporation) and analyzed using the UPLC AAA H-Class Application Kit (Waters Corporation, Milford, MA) according to manufacturer’s instructions. Amino acids were quantified using a Waters Acquity UPLC System with an AccQ-Tag Ultra C18 1.7 μm, 2.1 × 100 mm column and a photodiode detector array with a standard curve. Quality control checks (blanks and standards) were run every eight samples. For ornithine quantification from mouse chow, ornithine peaks were manually inspected and confirmed to be absent in the chow samples. All chemicals and reagents used were mass spectrometry grade. Principal component analysis (PCA) of the amino acid data was performed in R using the princomp function on scaled data. The heat map was generated in R using the heatmap.2 function in the gplots package (v. 3.2.0).

#### Transposon mutagenesis and screening

*E. coli* SM10 with the pBT20 plasmid containing a Himar1 transposon was grown on two LB agar plates with 75 mg/L carbenicillin overnight at 37°C for 18 hours, scraped up, resuspended in PBS, and normalized to OD_600_ = 160.^96^ An overnight culture of *A. baumannii astG*^*K76A, D147A*^Δ*gdhA::FRT* in 10 mL LB was centrifuged at 4000 × *g* for 5 minutes and the cell pellet was resuspended in PBS to OD_600_ = 50. The *E. coli* and *A. baumannii* were mixed in equal volumes and spotted onto an LB agar plate for mating. This plate was incubated at 37°C for 2.5 hours before being scraped up and pooled in 6.4 mL PBS. The resulting suspension was diluted 4X and 150 μL of this mixture was then spread onto LB agar plates with 20 mg/L chloramphenicol and 10 mg/L gentamicin. These plates were then incubated at 30°C for 40 hours. The colonies were velvet replica printed to M9 minimal media plates containing glutamate or succinate as the sole carbon source to identify any mutants that lost the ability to catabolize glutamate but retained growth on succinate. A total of 41,240 colonies were screened.

#### Shotgun metagenomics of human fecal samples

The data used here are publicly available in the NCBI SRA and as previously published.^90,91,123^ Human DNA isolation and shotgun metagenomic sequencing were performed as previously described.^90^ Briefly, DNA was extracted from feces and negative controls using DNeasy PowerSoil HTP 96 Kit (Qiagen) following the manufacturer’s instructions. Shotgun libraries were generated from 1 ng of DNA using the Nextera XT kit (Illumina). Libraries were sequenced on the Illumina HiSeq using 2 × 125 bp chemistry in High Output mode.

Shotgun metagenomic data were analyzed using Sunbeam v.2.0.1.^100^ The taxonomic count matrix from Kraken was subjected to quality filtering using the R package phyloseq.^101^ Taxa that were present in fewer than 25% of samples and less than 1000 cumulative reads were removed. Samples with less than 1 million reads were also removed from the dataset prior to analysis. Statistics on *A. baumannii* relative abundance between age groups and feeding type were performed using a Kruskal-Wallis test, and group comparisons were obtained using a Dunn’s post hoc test using the R package rstatix. P values displayed in figures were corrected for the false discovery rate.

To validate the presence of *A. baumannii* using metagenomic data, KrakenUniq was run on quality filtered shotgun metagenomic sequencing reads.^102^ The extract_reads script provided with KrakenUniq was used to gather reads that were identified as *A. baumannii* from each sample. Reads were then aligned using bwa to an *A. baumannii* pangenome that was assembled using all complete *A. baumannii* genomes available through the NCBI RefSeq database (733 genomes). Samples which had reads align to the *A. baumannii* pangenome and had an *A. baumannii* read count above zero following rarefaction were considered positive for *A. baumannii* in the subsequent analysis.

Shannon entropy scores were calculated using the estimate richness function in the phyloseq package.^101^ Statistics on Shannon entropy scores between age groups were performed using a Kruskal Wallis-test, and group comparisons were obtained using a Dunn’s post hoc test using the package rstatix. P values were corrected post hoc for the false discovery rate. Graphs were generated using the R package ggplot2.

### QUANTIFICATION AND STATISTICAL ANALYSES

All statistical analyses were performed in R v.4.0.2 or Prism v.10.1.1 (GraphPad). Statistical tests used are indicated in each figure legend. For bacterial enumeration in mouse experiments, approximate limits of detection (LOD) were calculated using the average sample mass within each experiment. Statistical details are provided in the figure legends. For bacterial culture experiments, *n* represents an independent culture from a single colony; for mouse experiments, *n* represents a sample from one animal; for human data, *n* represents a sample from one person. Figures were prepared in Adobe Illustrator 28.1.

## Supplementary Material

MMC1

MMC2

MMC3

SUPPLEMENTAL INFORMATION

Supplemental information can be found online at https://doi.org/10.1016/j.chom.2025.07.003.

## Figures and Tables

**KEY RESOURCES TABLE:** 

REAGENT or RESOURCE	SOURCE	IDENTIFIER

Bacterial and virus strains		

*Acinetobacter baumannii* ATCC 17978VU	ATCC^89^	LP486
*Acinetobacter baumannii* ATCC 17978VU *att::mTn7*	This study	LP526
*A. baumannii ΔastO* deletion strain used in intranasal infection	This study	LP720
*A. baumannii ΔastO ΔastA* deletion strain used in retroorbital infection	This study	LP516
*Acinetobacter baumannii* ATCC 17978VU *ΔastO::kan*	This study	LP814
*Acinetobacter baumannii* ATCC 17978VU *astA* ^*L125A H229A*^	This study	LP838
*Acinetobacter baumannii* ATCC 17978VU *astA* ^*L125A H229A*^*ΔastO::kan*	This study	LP872
*Acinetobacter baumannii* ATCC 17978VU *pWH1266-P*_*rpsA*_	This study	LP731
*Acinetobacter baumannii* ATCC 17978VU *ΔastO::kan pWH1266-P*_*rpsA*_	This study	LP732
*Acinetobacter baumannii* ATCC 17978VU *ΔastO::kan pWH1266-P*_*rpsA*_*-astO*	This study	LP1019
*Acinetobacter baumannii* ATCC 17978VU *ΔastO::kan pWH1266-P*_*rpsA*_*-astO(A. colistiniresistens)*	This study	LP1020
*Acinetobacter baumannii* ATCC 17978VU *astG* ^*K76A D147A*^	This study	LP840
*Acinetobacter baumannii* ATCC 17978VU *ΔgdhA::kan*	This study	LP1011
*Acinetobacter baumannii* ATCC 17978VU *astG* ^*K76A D147A*^ *ΔgdhA::kan*	This study	LP1050
*Acinetobacter baumannii* ATCC 17978VU *astG* ^*K76A D147A*^*ΔgdhA::FRT*	This study	LP1261
*Acinetobacter baumannii* ATCC 17978VU *ΔhutH::kan*	This study	LP1292
*Acinetobacter baumannii* ATCC 17978VU *ΔhutH::FRT*	This study	LP1300
*Acinetobacter baumannii* ATCC 17978VU *ΔhutH::FRT ΔastO::kan*	This study	LP1307
*Acinetobacter baumannii* ATCC 17978VU *ΔhutH::FRT att::mTn7*	This study	LP1301
*Acinetobacter baumannii* ATCC AYE	ATCC	LP13
*Acinetobacter baumannii* 0057	Robert Bonomo (Case Western Reserve University)	LP14
*Acinetobacter baumannii* ACICU	M. Stephen Trent (University of Georgia)	LP293
*Acinetobacter baumannii* ABUW AB5075	Colin Manoil (University of Washington)	LP345
*Acinetobacter nosocomialis*	Mario Feldman (Washington University at St. Louis)	LP116
*Acinetobacter baylyi* ADP1	ATCC	LP459
*Acinetobacter colistiniresistens*	DSMZ	LP697
*Acinetobacter gyllenbergii*	DSMZ	LP698
*Escherichia coli* K12	Maria Hadjifrangiskou (Vanderbilt University Medical Center)	LP235
*Escherichia coli* BW25113	Matthew Chapman (University of Michigan)	BW25113
*Pseudomonas aeruginosa* PAO1	Andrea Battistoni (University of Rome “Tor Vergata”)	LP346

Oligonucleotides		

GTTAAAAAGGATCGATCCTCTAGAGGATCCTATACAAATGAACCCGTTCTAC		*astO*_up_F
AGCTCCAGCCTACACGCTGCTTGTTCATCCTTTTG		*astO*_up_R
GAGGATATTCATATGGCGGAAATTACTATACATTTCAC		*astO*_dn_F
TGACCATGATTACGAATTCGAGCTCGGTACCATTTGGAGAAGTAAACCC		*astO*_dn_R
TGGTGAAGGTTCACGTATCTGGG		*astO*_up_up_F
CGGATCTTTGGTTTCAGCAGCAG		*astO*_dn_dn_R
GTGTAGGCTGGAGCTGCTTC		pKD4-FRTfrag_F
CATATGAATATCCTCCTTAGTTCCTATTC		pKD4-FRTfrag_R
GTTAAAAAGGATCGATCCTCTAGAATGTACTTACGACTGCAAAAG		*astA*_up_F
AATGCTGTACAGAGCT CACTAC		*astA*_L125A_R
AGTGAGCTCTGTACAG CATTTTTA		*astA*_L125A_F
TGTGGTGCCATTTTTCCAATCA		*astA*_H229A_R
AAAAATGGCACCACATACTTTGCC		*astA*_H229A_F
AATTCGAGCTCGGTACCTACAACTGTGTTACCAGCAAGT		*astA*_dn_R
TTATCAGGTATATCCTCGAGATGATGATTATTCGTTACATTGAAC		Com_*astO*_F
GGGCATCGGTCGACGGTACCATTAACAGCCATTCGAAAAT		Com_*astO*_R
TTATCAGGTATATCCTCGAGATGATGCTGATTCGTTATATCA		Com_*astO(A.colistiniresistens)*_F
GGGCATCGGTCGACGGTACCTCAATTTTTCTTTGGAATATTG		Com_*astO(A.colistiniresistens)*_R
GTTAAAAAGGATCGATCCTCTAGAGGGCGCAACTTGAGCTTTAAAGTG		*astG*_up_F
TGGTGCAGGTGGTGTTCGTTAC		*astG*_K76A_F
TAACGAACACCACCTGCACCAGGACCACGAGACAA		*astG*_K76A_R
ATCGGACCTCAAAAAGCAATTCCT		*astG*_D147A_F
AGCAGGAATTGCTTTTTGAGGT		*astG*_D147A_R
ACGAATTCGAGCTCGGTACCAAGGTAGTTTCCTCAGATTAG		*astG*_dn_R
AAAAGGATCGATCCTCTAGAGGATCCTTATTCTTAACTTTAATGCAGACG		*gdhA*_up_F
AGGAACTAAGGAGGATATTCATATGAATATAGAAATAAAAAAATCCGATGCA		*gdhA*_up_R
ACTTCGAAGCAGCTCCAGCCTACACTTTAAAATGCATCATTTAGCAGC		*gdhA*_dn_F
ATGATTACGAATTCGAGCTCGGTACGAGCTGCTTTTCCAACTTTT		*gdhA*_dn_R
GTTTTGCGGGGTATACCA		*gdhA*_up_up_F
TGCTTGTGCCGATAACATA		*gdhA*_dn_dn_R
AAAAGGATCGATCCTCTAGAGGATCTGGTGTACGTCCAGACA		*hutH*_up_F
ACTTCGAAGCAGCTCCAGCCTACACGTTGGCTTCCTAATTTTTTGCA		*hutH*_up_R
AGGAACTAAGGAGGATATTCATATGTTGATAAAACGGTCACATGGAG		*hutH*_dn_F
ATGATTACGAATTCGAGCTCGGTACGTACAACATACGGCCTGC		*hutH*_dn_R
TTACTGCTGGTTTAGGC		*hutH*_up_up_F
GTGCCTTAATTTCAGCAGTA		*hutH*_dn_dn_R
AGAGTTTGATYMTGGCTCAG		16s-CS1_27F-YM
AGAATTTGATCTTGGTTCAG		16s-CS1_27F-Chl
AGAGTTTGATCCTGGCTTAG		16s-CS1_27F-Bor
AGGGTTCGATTCTGGCTCAG		16-CS1_27F-Bif
AGAGTTCGATCCTGGCTCAG		16s- CS1_27F-Ato
ATTACCGCGGCTGCTGG		16s- 534R
ATAACTGCACCCACTTCCCA		Il10_F
GGGCATCACTTCTACCAGGT		Il10_R
CTGCAAGAGACTTCCATCCAG		Il6_F
AGTGGTATAGACAGGTCTGTTGG		Il6_R
ACATTTGTTCCAAGCTCCAGGGC		Lcn2_F
CATGGCGAACTGGTTGTAGTCCG		Lcn2_R
TTGGGTCTTGTTCACTCCACGG		Nos2_F
CCTCTTTCAGGTCACTTTGGTAGG		Nos2_R
TGTCCTCAGTTTGTGCAGAATATAAA		S1008a_F
TCACCTCGCAAGGAACTCC		S1008a_R
ATGGCCTCCCTCTCATCAGT		Tnfa_F
CTTGGTGGTTTGCTACGACG		Tnfa_R
GCTCCAGAAGGCCCTCAGA		Il17a_F
AGCTTTCCCTCCGCATTGA		Il17a_R
GGCTGTATTCCCCTCCATCG		Actb_F
CCAGTTGGTAACAATGCCATGT		Actb_R
GTGTAGGCTGGAGCTGCTTC		Δ*hutH*::FRT_F
CCCAGTTCCAATTGCTGAGCCC		Δ*hutH*::FRT_R
GCAAGCGTGGCTTGTGTAGAGC		*hutH*_F
CCAGAGAAACCACGTGCCAAGC		*hutH*_R

Biological samples		

Human infant fecal samples	This study	N/A

Chemicals, peptides, and recombinant proteins		

LB Agar, Miller	Fisher	Cat# BP1425
LB Broth, Miller	BD	Cat# 244610
D-Sucrose	Fisher	Cat# BP220
Kanamycin sulfate	VWR	Cat#75856-686
Carbenicillin	Fisher	Cat# BP26485
Chloramphenicol	Fisher	Cat# BP904100
Gentamicin sulfate	Fisher	Cat# MT61098RF
Ampicillin sodium salt	Sigma	Cat# A9518
Streptomycin sulfate salt	Sigma	Cat# S6501
Vancomycin hydrochloride USP	Mylan	NDC 72078-066-99
Ammonium chloride	Fisher	Cat# A702
L-arginine hydrochloride	VWR	Cat# A1337
L-ornithine hydrochloride	Fisher	Cat# AAA1211122
Sodium succinate hexahydrate	Fisher	Cat# AA3338630
Monosodium L-glutamate	Spectrum	Cat# G1038
L-histidine monohydrochloride monohydrate	Thermo Fisher	Cat# A17627.18
Molecular Biology Grade Water	Fisher	Cat#46-000-CV
Phosphate Buffered Saline	Fisher	Cat# MT21-040-CV
Tri-Reagent	Molecular Research Center	Cat# TR 118
1-bromo-3-chloropane	Sigma	Cat# B9673
Isopropanol	Sigma	Cat# I9516
40% 29:1 bisacrylamide/acrylamide	BioRad	Cat# 1610146
Paraformaldehyde	Electron Microscopy Sciences	Cat# 15713
VA-044	FujiFilm	Cat# LB-VA044-50GS
Glacial acetic acid	Fisher	Cat# A38C-212
Methanol	Fisher	Cat# A452-4
Chloroform	Sigma Aldrich	Cat# 650498
DAPI	Thermo Fisher	Cat# D21490
Wheat germ agglutinin [WGA] – Alexa Fluor 647	Thermo Fisher	Cat# W32466
Fluorescent DNA hairpins for FICR	Molecular Instruments	Amplifiers B1-546 and B4-488

Critical commercial assays		

QIAamp PowerFecal Pro DNA Kit	Qiagen	Cat# 51804
QIAprep Spin Miniprep Kit	Qiagen	Cat# 27106
Promega GoTaq PCR Green Mix	Promega	Cat# M7123
NEB DNA HIFI Assembly	NEB	Cat# E2621L
Q5^®^ High-Fidelity 2X Master Mix	NEB	Cat# M0492S
Breathe-Easy sealing membrane	Sigma	Cat# Z380059
TURBO DNA-free kit	Invitrogen	Cat# AM1907
High-Capacity cDNA Reverse Transcription Kit	Applied Biosystems	Cat# 4368814
Fast SYBR Green Master Mix	Applied Biosystems	Cat# 4385612
AccQ-Tag Ultra Amino Acid Derivatization Kit	Waters Corporation	Cat# 186003836
UPLC AAA H-Class Application Kit	Waters Corporation	Cat# 176002983
repliQa HiFi ToughMix	QuantaBio	Cat# 95200
Access Array for barcoding system	Fluidigm	Cat# 100-4876
DNeasy PowerSoil HTP 96 Kit	Qiagen	Cat# 12955
Nextera XT DNA Library Preparation Kit	Illumina	Cat# FC-131-1096

Deposited data		

Human infant metagenomics from the IGRAM study	Rimal et al.^90^	NCBI: PRJNA1042647
Human infant metagenomics from the IGRAM study	Soto Ocaña and Friedman et al.^91^	NCBI: PRJNA1145027
Human infant metagenomics from the IGRAM study	Soto Ocaña and Friedman et al.^91^	NCBI: PRJNA1106565
Human infant metagenomics from the IGRAM study	This study	NCBI: PRJNA1173239
Mouse 16S rRNA gene sequencing	This study	NCBI: PRJNA1178140
Whole genome sequencing of *A. baumannii* 17978VU att::mTn7 (LP526) and *ΔastO::kan* (LP814)	This study	NCBI: PRJNA1173193

Experimental models: Organisms/strains		

C57BL/6J	Jackson Laboratory	Cat# 000664
Swiss Webster- Charles River	Charles River	Cat# 024
Swiss Webster Hsd:ND4- Inotiv	Inotiv	Cat# 032
Germ-free Swiss-Webster	Behnsen Lab	N/A

Recombinant DNA		

pFLP2	Hoang et al.^92^	N/A
pKD4	Datsenko and Wanner^93^	N/A
pKNOCK-mTn7-Amp	Carruthers et al.^94^	N/A
pAT03	Tucker et al.^95^	N/A
pBT20	Kulasekara et al.^96^	N/A
pWH1266	Hunger et al.^97^	N/A
pWH1266-*rpsAp*	Palmer et al.^98^	pLDP29
pWH1266-*rpsAp-astO*	This study	pLDP171
pWH1266-*rpsAp-astO (A. colistiniresistens)*	This study	pLDP181
pFLP2-*ΔastO::Kn*	This study	pLDP94
pFLP2-*astA1*^L125A H229A^	This study	pLDP216
pFLP2-*astG*^K76A D147A^	This study	pLDP218
pFLP2-*ΔgdhA::Kn*	This study	pLDP284
pFLP2-*ΔhutH::Kn*	This study	pLDP364

Software and algorithms		

Graphpad Prism v 10.1.1	GraphPad Software	https://www.graphpad.com
Imaris	Oxford Instruments	https://imaris.oxinst.com/
Fiji	ImageJ	https://imagej.net/software/fiji/
Mash v 2.34	Mash	https://github.com/marbl/Mash/releases
Orthofinder v 2.5.4	Emms and Kelly^99^	https://github.com/davidemms/OrthoFinder/releases
R v4.0.2	The R Foundation	https://www.r-project.org/
Sunbeam v 2.0.1	Clarke et al.^100^	https://github.com/sunbeam-labs/sunbeam
Phyloseq v 1.52.0	McMurdie and Holmes^101^	https://github.com/joey711/phyloseq
Rstatix v 0.7.2	N/A	https://rpkgs.datanovia.com/rstatix/
KrakenUniq v 1.0.4	Breitwieser et al.^102^	https://github.com/fbreitwieser/krakenuniq
Gplots v 3.2.0	N/A	https://github.com/talgalili/gplots

Other		

Bullet Blender NAVY lysis tubes 1.5 mL	NextAdvance	NAVYE5
AccQ-Tag Ultra C18 1.7 μm, 2.1 × 100 mm column	Waters	186003837
LM-485 mouse/rat sterilizable diet (irradiated)	Teklad	Cat# 7912
Autoclaved 5L79	PMI Nutrition International	Cat# 5L79
NIH-31 Modified Open (irradiated)	Teklad	Cat# 7913

## Highlights

- Pathogenic *Acinetobacter* spp. encode AstO for ornithine catabolism
- *A. baumannii* requires AstO to compete with the microbiota in the mouse gut
- Carbon sources preferred over ornithine rescue the Δ*astO* mutant in mice
- Diet influences *A. baumannii* gut carriage in human infants
