# Supplementary material for: Amino acid competition shapes Acinetobacter baumannii gut carriage
Source: Cell Host Microbe. Author manuscript; Available in PMC 2025 Aug 24. (PMC12375414; doi:10.1016/j.chom.2025.07.003)
Supplement: MMC1 [file NIHMS2101725-supplement-MMC1.pdf]

Cell Host & Microbe, Volume *33*

Supplemental information

Amino acid competition shapes *Acinetobacter baumannii* gut carriage

Xiaomei Ren, R. Mason Clark, Dziedzom A. Bansah, Elizabeth N. Varner, Connor R. Tiffany, Kanchan Jaswal, John H. Geary, Olivia A. Todd, Jonathan D. Winkelman, Elliot S. Friedman, Riley N. Jarrett, Babette S. Zemel, Gary D. Wu, Joseph P. Zackular, William H. DePas, Judith Behnsen, and Lauren D. Palmer

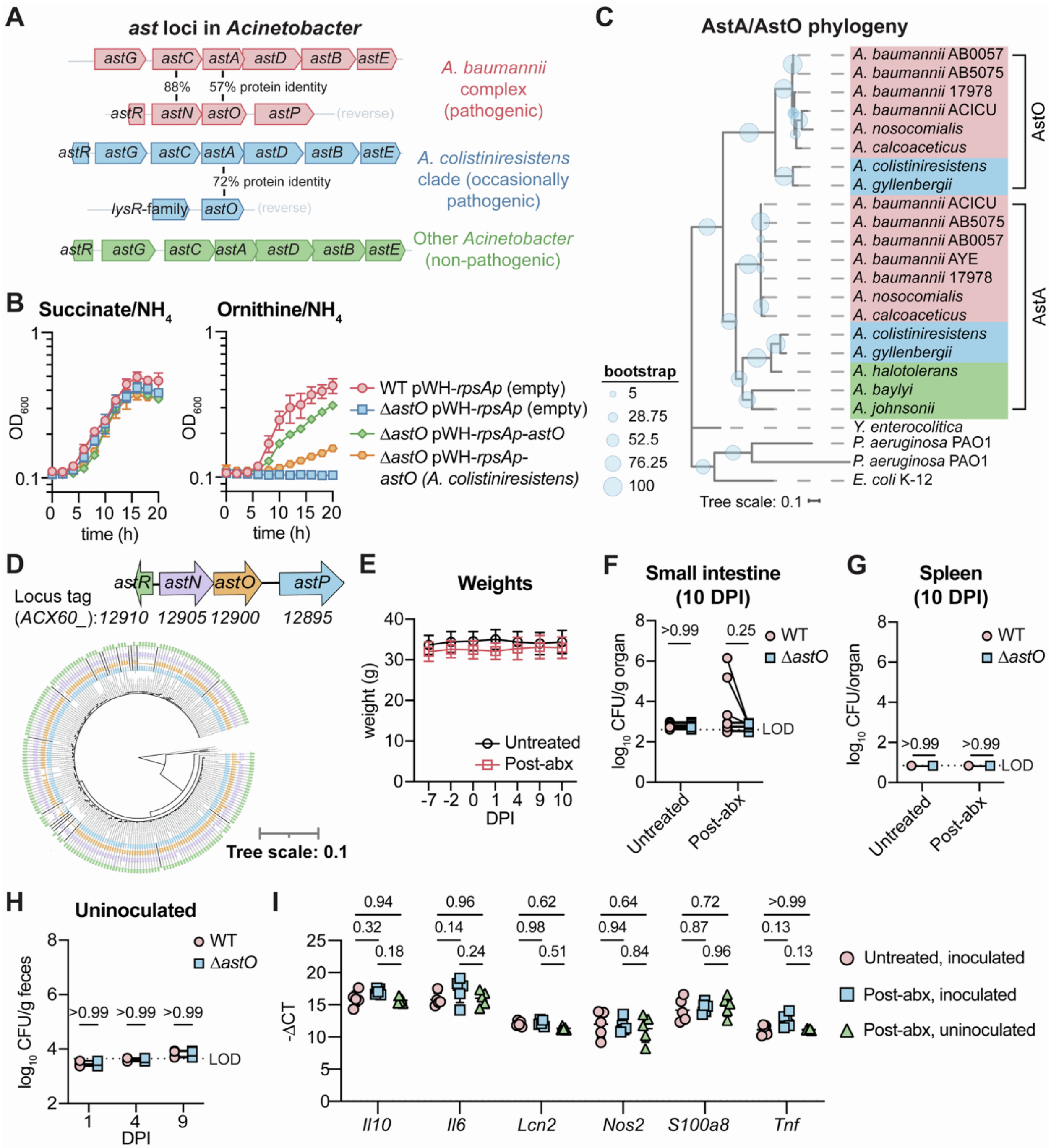

Fig S1. Evidence of evolution at the second *ast* locus in *Acinetobacter* species and requirement for *A. baumannii* gut colonization.

Related to Figure 1

(**A**) *ast* loci in *Acinetobacter* clades. Members of the *abc* clade such as *A. baumannii* 17978 encode the second *ast* locus with *astR*, and *astNOP* on the negative strand (red). Members of the *A. colistiniresistens* clade encode the *astGCADBE* locus with *astR* divergently transcribed; the second *ast* locus has only *astO* and a divergent LysR-family regulator gene (blue). Other non-pathogenic *Acinetobacter* spp. only one *ast* locus with *astR* and *astGCABDE* (green).

(**B**) Growth of *A. baumannii* 17978 WT pWH (empty vector), Δ*astO* pWH, Δ*astO* pWH-*astO* (*A. baumannii*) and Δ*astO* pWH-*astO* (*A. colistiniresistens*) grown in M9 minimal media with

succinate or ornithine as the sole carbon source. Growth was monitored by OD_{600} measurement (n = 3, mean ± SD, experiments were performed at least twice with similar results).
(**C**) Phylogenetic tree of AstA and AstO proteins.
(**D**) Second *ast* locus genes and their corresponding copy numbers mapped to an *A. baumannii* species phylogenetic tree generated from 233 de-duplicated published *A. baumannii* and *Acinetobacter* genomes (see Table S1).
(**E**) Weight of mice in Fig. 1G-H (n = 10, mice combined from 2 independent experiments, mean ± SD).
(**F**) *A. baumannii* 17978 CFU in the small intestine from mice shown in Fig. 1G-H (n = 10, mice combined from 2 independent experiments, *p* by Wilcoxon test with Holm-Sidak's multiple comparisons).
(**G**) *A. baumannii* 17978 CFU in the spleen from mice shown in Fig. 1G-H (n = 10, mice combined from 2 independent experiments; *p* by Wilcoxon test with Holm-Sidak's multiple comparisons).
(**H**) *A. baumannii* CFU enumerated from uninoculated mice (n = 5, *p* by two-way ANOVA with Sidak's multiple comparisons).
(**I**) qRT-PCR of genes encoding inflammatory proteins quantified from mice cecum tissue in Figures 1G-H and S1H. -ΔCT compared to *Actb* (n = 5, *p* by two-way ANOVA with Tukey's multiple comparisons).
Lines connect CFU enumerated from the same mouse. OD_{600}, optical density at 600 nm; DPI, days post inoculation; CFU, colony forming units; LOD, approximate limit of detection; post-abx, post antibiotics.

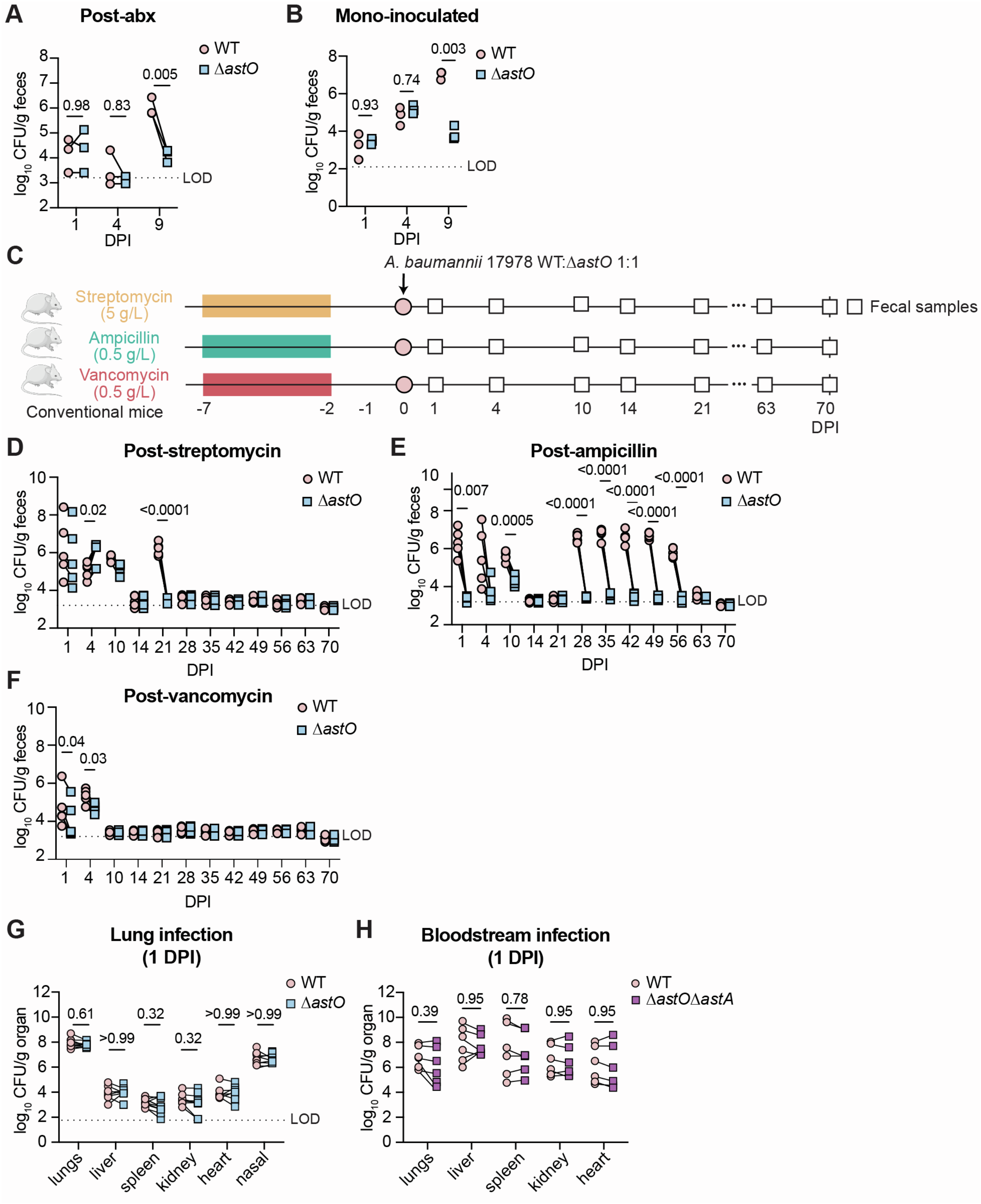

Fig S2. *A. baumannii* AstO is required for gut colonization after multiple antibiotics but not for *A. baumannii* lung or bloodstream infection.

Related to Figure 1

(**A**) Male C57BL/6 mice were inoculated by orogastric gavage with 1:1 *A. baumannii* 17978 WT and Δ*astO* after antibiotic pretreatment with gentamicin as in Figure 1F (n = 3, *p* by Wilcoxon test with Holm-Sidak's multiple comparisons).
(**B**) Female Swiss Webster mice pretreated with gentamicin were mono-inoculated with *A. baumannii* 17978 WT or Δ*astO* (n = 3, *p* by two-way ANOVA with Sidak's multiple comparisons).
(**C**) Experimental setup to compare *A. baumannii* gut colonization following treatment with different antibiotics.
(**D-F**) Female Swiss Webster mice were inoculated with 1:1 *A. baumannii* 17978 WT and Δ*astO* after pretreatment with the designated antibiotic (n = 5, *p* by two-way ANOVA with Sidak's multiple comparisons, $p < 0.05$ are displayed).
(**G**) Female C57BL/6 mice were intranasally inoculated with 1:1 *A. baumannii* 17978 WT and Δ*astO*. CFU enumeration at 1 DPI (n = 10, *p* by Wilcoxon test with Holm-Sidak's multiple comparisons).
(**H**) Male C57BL/6 mice were retroorbitally inoculated with 1:1 *A. baumannii* 17978 WT and Δ*astA*Δ*astO*. CFU were enumerated at 1 DPI (n = 6 mice combined from 2 independent experiments, *p* by Wilcoxon test with Holm-Sidak's multiple comparisons).
Lines connect CFU enumerated from the same mouse. DPI, days post inoculation; CFU, colony forming units; LOD, approximate limit of detection.

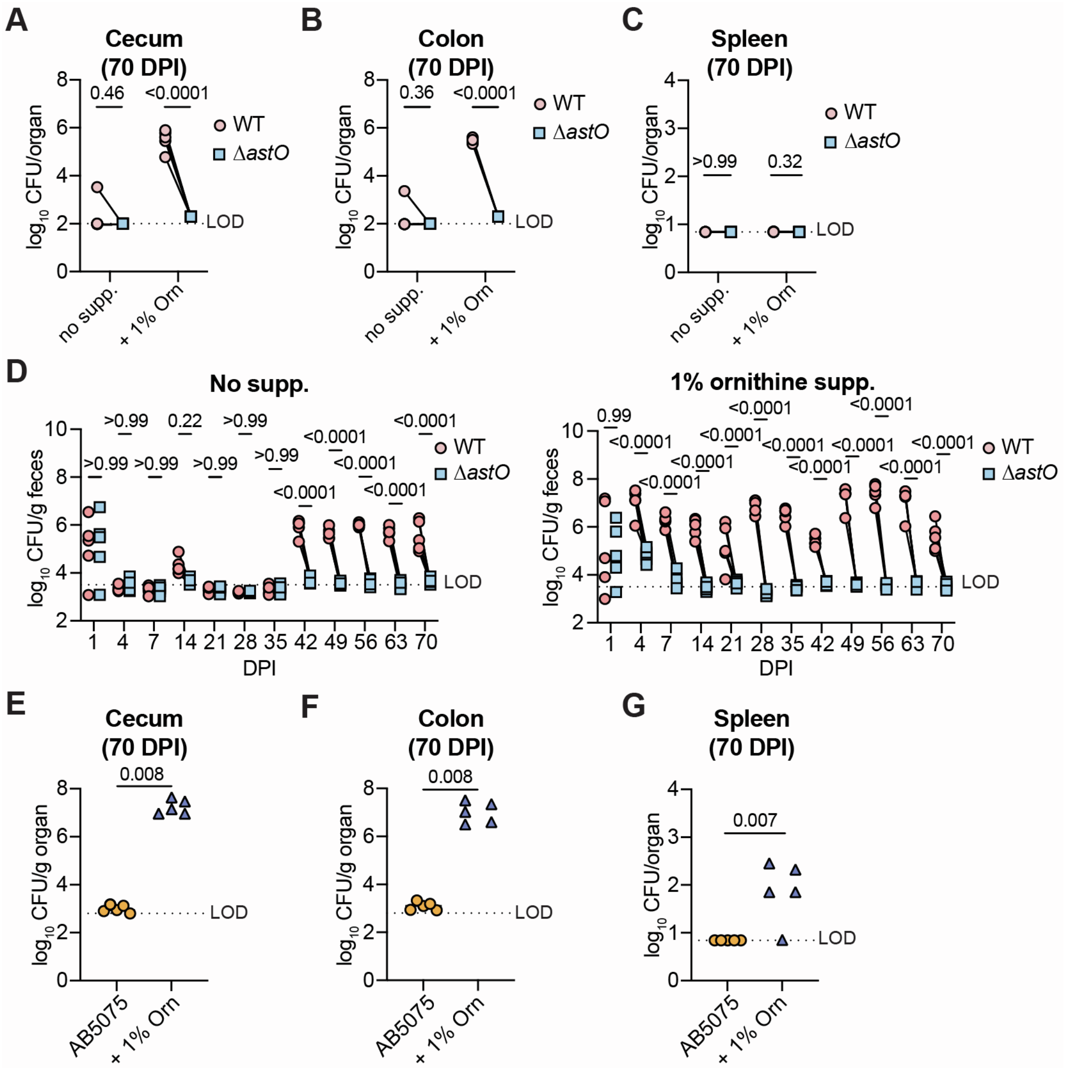

Figure S3 Supplemental ornithine promotes long-term *A. baumannii* gut colonization
Related to Figure 2
(**A-C**) Mice (from Figure 2B) were euthanized at 70 DPI and *A. baumannii* CFU were enumerated from the cecum, colon and spleen (n = 5, *p* by two-way ANOVA with Sidak's multiple comparisons).
(**D**) Female C57BL/6J mice were fed NIH-31 Modified Open Formula chow (Teklad 7913). Experimental design as in Figure 2A. *A. baumannii* 17978 CFU from fecal samples at the indicated DPI. (n = 5, *p* by two-way ANOVA with Sidak's multiple comparisons; experiments were repeated twice with similar results).
(**E-G**) Mice (from Figure 2D) were euthanized at 70 DPI and *A. baumannii* 5075 CFU were enumerated from the cecum, colon and spleen (n = 5, *p* by two-way ANOVA with Sidak's multiple comparisons).
Lines connect CFU enumerated from the same mouse. DPI, days post infection; CFU, colony forming units; LOD, approximate limit of detection; supp., supplementation.; Orn, ornithine.

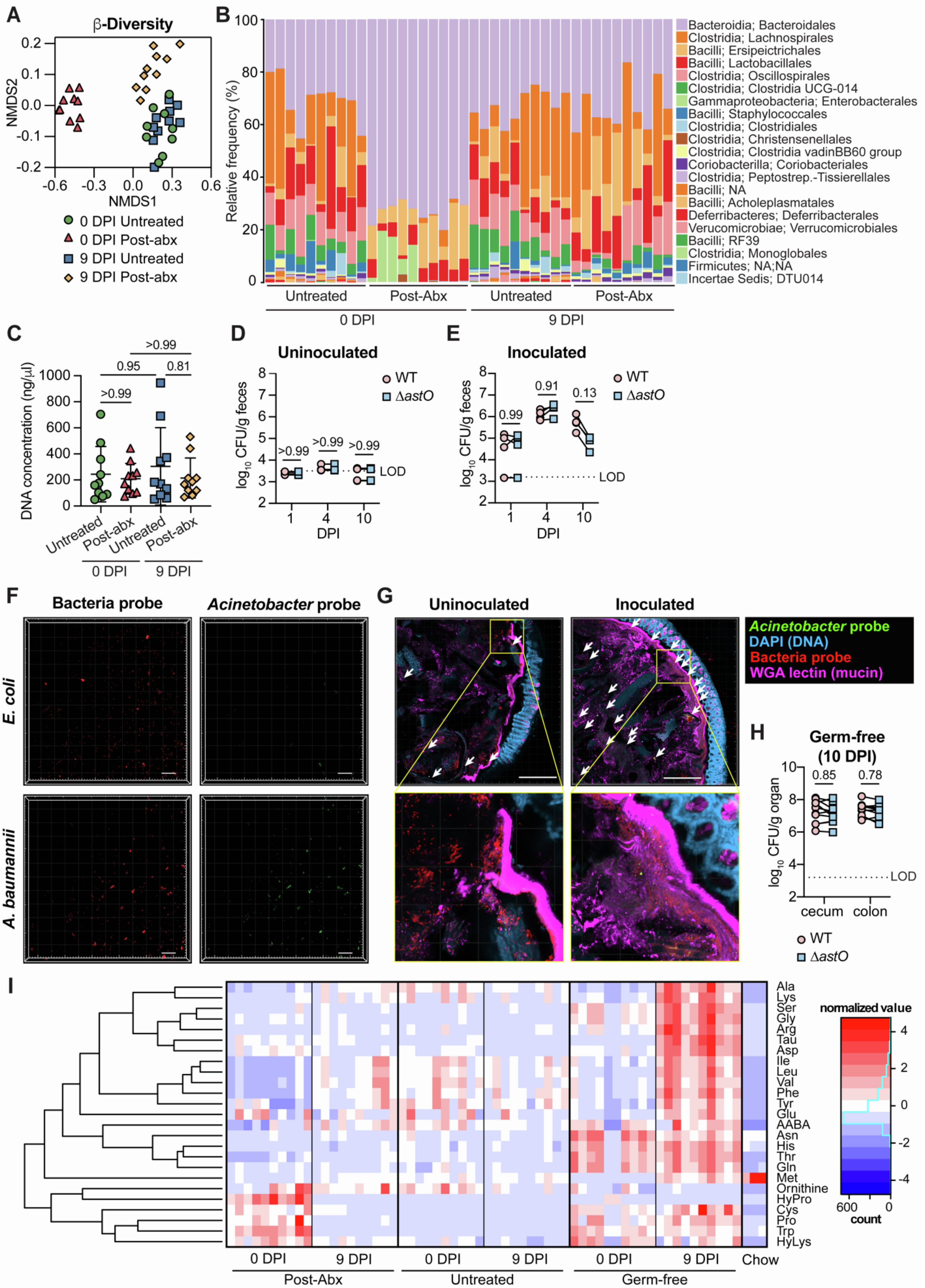
A
β-Diversity
NMDS1
NMDS2
0 DPI Untreated
0 DPI Post-abx
9 DPI Untreated
9 DPI Post-abx
B
Relative frequency (%)
Bacteroidia; Bacteroidales
Clostridia; Lachnospirales
Bacilli; Ersipeictrichales
Bacilli; Lactobacillales
Clostridia; Oscillospirales
Clostridia; Clostridia UCG-014
Gammaproteobacteria; Enterobacterales
Bacilli; Staphylococcales
Clostridia; Clostridiales
Clostridia; Christensenellales
Clostridia; Clostridia vadinBB60 group
Coriobacterilla; Coriobacteriales
Clostridia; Peptostrep.-Tissierellales
Bacilli; NA
Bacilli; Acholeplasmatales
Deferribacteres; Deferribacterales
Verucomicrobiae; Verrucomicrobiales
Bacilli; RF39
Clostridia; Monoglobales
Firmicutes; NA;NA
Incertae Sedis; DTU014
Untreated
Post-Abx
Untreated
Post-Abx
0 DPI
9 DPI
C
DNA concentration (ng/μl)
>0.99
0.95
0.81
>0.99
Untreated
Post-abx
Untreated
Post-abx
0 DPI
9 DPI
D
Uninoculated
log10 CFU/g feces
WT
ΔastO
>0.99
>0.99
>0.99
LOD
DPI
E
Inoculated
log10 CFU/g feces
WT
ΔastO
0.99
0.91
0.13
LOD
DPI
F
Bacteria probe
Acinetobacter probe
E. coli
A. baumannii
G
Uninoculated
Inoculated
Acinetobacter probe
DAPI (DNA)
Bacteria probe
WGA lectin (mucin)
H
Germ-free (10 DPI)
log10 CFU/g organ
0.85
0.78
LOD
cecum
colon
WT
ΔastO
I
normalized value
count
Ala
Lys
Ser
Gly
Arg
Tau
Asp
Ile
Leu
Val
Phe
Tyr
Glu
AABA
Asn
His
Thr
Gln
Met
Ornithine
HyPro
Cys
Pro
Trp
HyLys
0 DPI
9 DPI
0 DPI
9 DPI
0 DPI
9 DPI
Chow
Post-Abx
Untreated
Germ-free

Figure S4 Microbiota diversity, *Acinetobacter* imaging, and amino acid metabolome
Related to Figure 3
(**A**) β-diversity NMDS plot of 16S rRNA gene profiling at 0 and 9 DPI in the feces of untreated and post-abx female Swiss Webster mice inoculated with *A. baumannii* 17978 WT and Δ*astO* and shown in Fig. 2B-C (n = 10).
(**B**) Relative abundance of bacterial ASV identified by 16S rRNA gene sequencing at 0 and 9 DPI in the feces of untreated and post-abx female Swiss Webster mice shown in Fig. 2B-C (n = 10).
(**C**) DNA concentration of gDNA extracted from feces on 0 and 9 DPI from mice in Figure 1F (n = 10, mean ± SD, *p* by one-way ANOVA with Sidak's multiple comparisons).
(**D-E**) Post-gentamicin female Swiss Webster mice were uninoculated or inoculated with *A. baumannii* 17978 WT and Δ*astO*. CFU were enumerated from fecal samples at 1, 4, and 9 DPI (n = 4, *p* by two-way ANOVA with Sidak's multiple comparisons). Samples were used for MiPACT-HCR imaging shown in Figure 3B and Figure S4G.
(**F**) MiPACT-HCR imaging of bacterial cultures to assess specificity of anti-*Acinetobacter* probe. Scale bar is 50 μm; green, anti-*Acinetobacter* HCR probe Aci16s 729; red, general bacterial HCR probe eub338, blue, DAPI; magenta,
(**G**) MiPACT-HCR imaging of colons from mice in Figure S4D-E to assess localization of anti-*Acinetobacter* probe Aci16s 729. Scale bar is 500 μm; green, anti-*Acinetobacter* HCR probe (highlighted with white arrows); red, general bacterial HCR probe eub338; blue, DAPI; magenta, WGA lectin.
(**H**) Germ-free mice were euthanized at 10 DPI and CFU were enumerated from the cecum and colon (n = 10, *p* by two-way ANOVA with Sidak's multiple comparisons).
(**I**) Heat-map of amino acid metabolome in chow and feces from untreated, post-abx, and germ-free mice from Figure 1G-H and Figure 3C. Tree on left shows hierarchical clustering of amino acids.
Lines connect CFU of strains enumerated from the same mouse. NMDS, non-metric multidimensional scaling; DPI, days post infection; Abx, antibiotics; CFU, colony forming units; ASV, amplicon sequence variants; LOD, approximate limit of detection; MiPACT-HCR, microbial identification after passive clarity technique via hybridization chain reaction; AABA, α-aminobutyric acid; HyLys, hydroxylysine; HyPro, hydroxyproline.

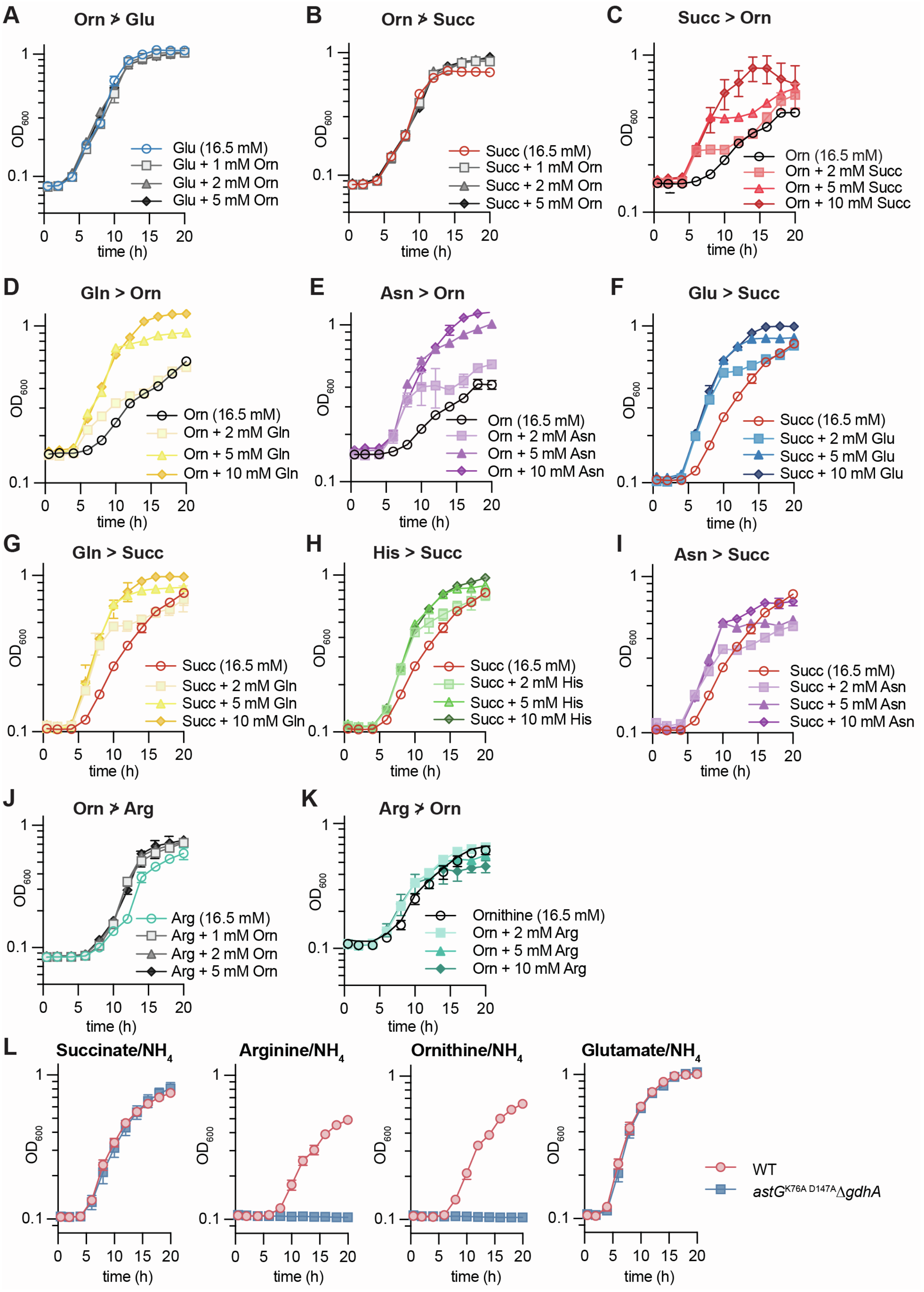
A
Orn ≯ Glu
Glu (16.5 mM)
Glu + 1 mM Orn
Glu + 2 mM Orn
Glu + 5 mM Orn
B
Orn ≯ Succ
Succ (16.5 mM)
Succ + 1 mM Orn
Succ + 2 mM Orn
Succ + 5 mM Orn
C
Succ > Orn
Orn (16.5 mM)
Orn + 2 mM Succ
Orn + 5 mM Succ
Orn + 10 mM Succ
D
Gln > Orn
Orn (16.5 mM)
Orn + 2 mM Gln
Orn + 5 mM Gln
Orn + 10 mM Gln
E
Asn > Orn
Orn (16.5 mM)
Orn + 2 mM Asn
Orn + 5 mM Asn
Orn + 10 mM Asn
F
Glu > Succ
Succ (16.5 mM)
Succ + 2 mM Glu
Succ + 5 mM Glu
Succ + 10 mM Glu
G
Gln > Succ
Succ (16.5 mM)
Succ + 2 mM Gln
Succ + 5 mM Gln
Succ + 10 mM Gln
H
His > Succ
Succ (16.5 mM)
Succ + 2 mM His
Succ + 5 mM His
Succ + 10 mM His
I
Asn > Succ
Succ (16.5 mM)
Succ + 2 mM Asn
Succ + 5 mM Asn
Succ + 10 mM Asn
J
Orn ≯ Arg
Arg (16.5 mM)
Arg + 1 mM Orn
Arg + 2 mM Orn
Arg + 5 mM Orn
K
Arg ≯ Orn
Ornithine (16.5 mM)
Orn + 2 mM Arg
Orn + 5 mM Arg
Orn + 10 mM Arg
L
Succinate/NH4
Arginine/NH4
Ornithine/NH4
Glutamate/NH4
WT
astG K76A D147A ΔgdhA
OD600
time (h)

Figure S5 ***A. baumannii*** **prefers other amino acids over ornithine as carbon sources.**
Related to Figure 4
(**A-K**) *A. baumannii* 17978 WT was grown in M9 media containing 16.5 mM carbon source only and indicated additions as carbon sources. Growth was monitored by OD_{600} measurement for 20 h (n = 3, mean ± SD, experiments were performed at least twice with similar results). The hierarchy of *A. baumannii* carbon source preference shown is Glu, Gln, Asn, His > Succ > Arg, Orn.
(**L**) *A. baumannii* 17978 WT and $astG^{K76A\ D147A}\Delta gdhA$ double mutant were grown in M9 media containing 16.5 mM of the indicated carbon source (n = 3, mean ± SD, experiments were performed at least twice with similar results).
OD_{600}, optical density at 600 nm; Orn, ornithine; Succ, succinate.

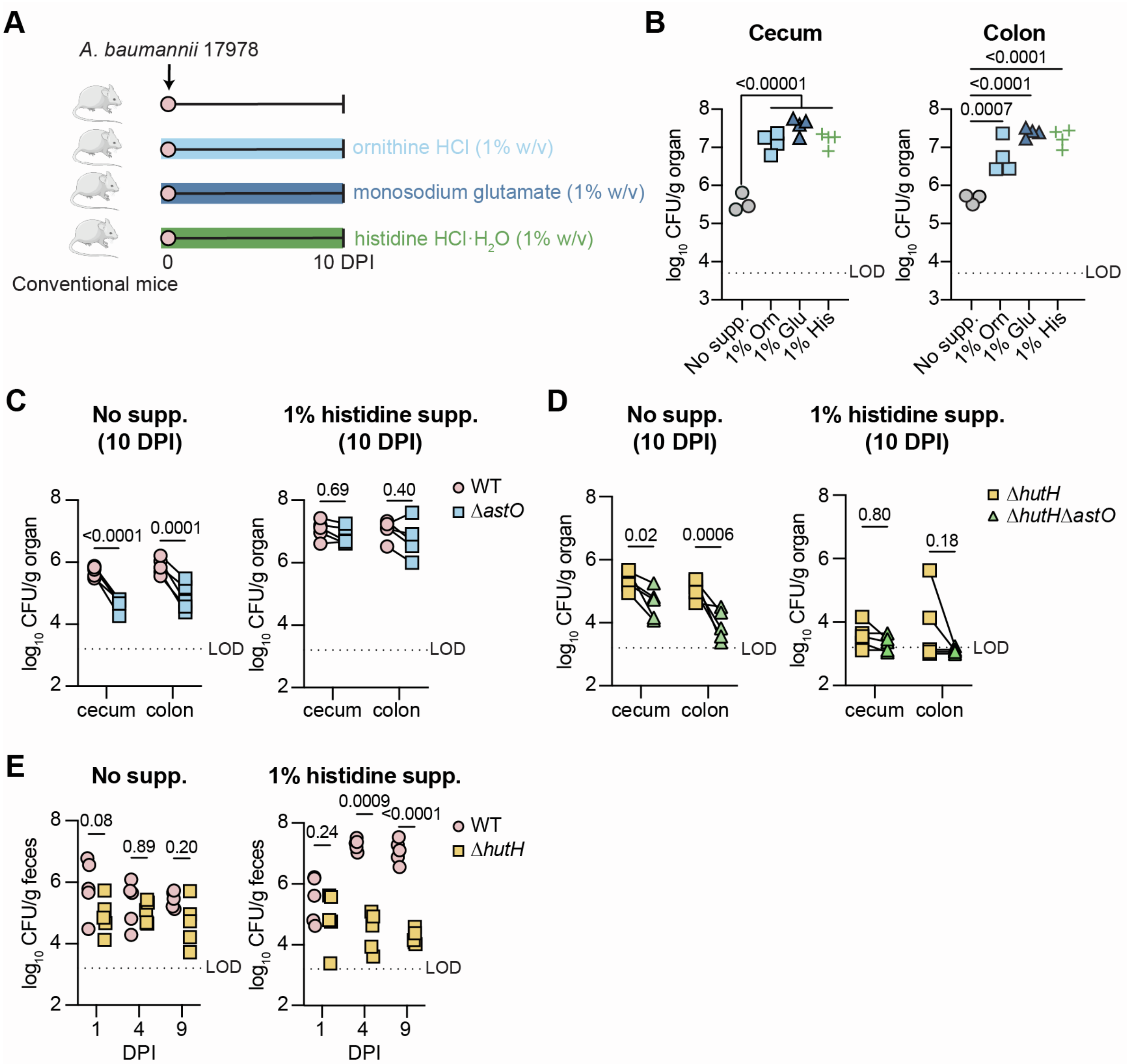

Figure S6. Supplemental histidine supports *A. baumannii* gut colonization when HutH is present.

Related to Figure 5.

(**A**) Experimental setup to compare *A. baumannii* gut colonization in antibiotic untreated mice following supplementation with ornithine, glutamate, or histidine.

(**B**) Mice were euthanized at 10 DPI and *A. baumannii* CFU were enumerated from the cecum and colon (n = 5, *p* by two-way ANOVA with Sidak's multiple comparisons).

(**C**) Mice (from Figure 5D-E) were euthanized at 10 DPI and *A. baumannii* CFU were enumerated from the cecum and colon (n = 5, *p* by one-way ANOVA with Dunnett's multiple comparisons).

(**D**) Mice (from Figure 5F-G) were euthanized at 10 DPI and *A. baumannii* CFU were enumerated from the cecum and colon (n = 5, *p* by two-way ANOVA with Sidak's multiple comparisons).

(**E**) *A. baumannii* WT and Δ*hutH* CFU were enumerated from feces (from Figure 5D, Figure 5F) (n=5, *p* by two-way ANOVA with Sidak's multiple comparisons).

Lines connect CFU enumerated from the same mouse. DPI, days post infection; CFU, colony forming units; supp., supplementation; LOD, approximate limit of detection; Orn, ornithine.
